# Progress in Multiscale Modeling of Silk Materials

**DOI:** 10.1021/acs.biomac.4c01122

**Published:** 2024-10-22

**Authors:** Harry D. A. Brough, David Cheneler, John G. Hardy

**Affiliations:** † Department of Chemistry, Lancaster University, Lancaster LA1 4YB, United Kingdom; ‡ School of Engineering, Lancaster University, Lancaster LA1 4YW, United Kingdom; § Materials Science Lancaster, Lancaster University, Lancaster, LA1 4YW, United Kingdom

## Abstract

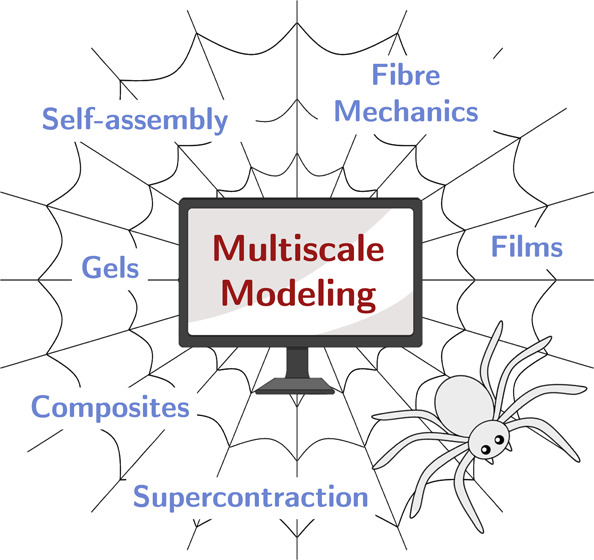

As a result of their hierarchical structure and biological processing, silk fibers rank among nature’s most remarkable materials. The biocompatibility of silk-based materials and the exceptional mechanical properties of certain fibers has inspired the use of silk in numerous technical and medical applications. In recent years, computational modeling has clarified the relationship between the molecular architecture and emergent properties of silk fibers and has demonstrated predictive power in studies on novel biomaterials. Here, we review advances in modeling the structure and properties of natural and synthetic silk-based materials, from early structural studies of silkworm cocoon fibers to cutting-edge atomistic simulations of spider silk nanofibrils and the recent use of machine learning models. We explore applications of modeling across length scales: from quantum mechanical studies on model peptides, to atomistic and coarse-grained molecular dynamics simulations of silk proteins, to finite element analysis of spider webs. As computational power and algorithmic efficiency continue to advance, we expect multiscale modeling to become an indispensable tool for understanding nature’s most impressive fibers and developing bioinspired functional materials.

## 1 Introduction

The production of cocoons by domesticated *Bombyx mori* silkworms has been harnessed by humans for thousands of years to supply strong, lustrous and biocompatible silk fibers for textiles and surgical sutures.^1−4^ Silk fibers are also spun by other animals such as ants, bees, caddisflies and spiders, and are used for diverse ecological functions.^5^ The dragline silks of some spiders have remarkable mechanical properties, with the tensile strength of steel, higher toughness than Kevlar and extreme extensibility,^6,7^ as demonstrated in Table 1. These properties have inspired numerous applications for spider silk, from tissue scaffolds to bulletproof vests and artificial muscles.^8−11^

**Table 1:** Mechanical Properties of Orb-Weaver Dragline Silks and Comparable Materials^6,7^

Material	Strength/GPa	Toughness/MJ m^–3^	Extensibility
Orb-weaver dragline silk	0.7–1.6	110–350	0.17–0.52
*Bombyx mori* cocoon silk	0.6	70	0.18
Elastin	0.002	1.6	1.5
Kevlar 49	3.6	50	0.027
High-tensile steel	1.5	6	0.008
Carbon fiber	4.0	25	0.013
Nylon	0.9	80	0.18
Synthetic rubber	0.05	100	8.5

Unfortunately, the territorial and cannibalistic nature of spiders makes large scale silk farming impossible. Routes toward synthetic spider silks via genetic engineering^12−14^ and chemical synthesis^15−17^ have been explored, but reproducing the mechanical properties of naturally spun fibers has been challenging due to the inherent complexity of structure–function relationships in natural silks and the fiber spinning process *in vivo*. Our understanding of these aspects has improved substantially over the past decade, in large part due to rapid advancements in computational modeling, and fibers that compete with naturally spun spider silk have recently been produced.^18,19^

### 1.1 Structure of Silk Fibers

Silk fibers are made of fibroin proteins, which are chains of amino acids encoded by genes.^4^ Amino acids self-assemble into folded secondary structures through hydrogen bonding, the most common in biology being α-helices, where hydrogen bonds link amino acids four residues apart in a spiral, and β-sheets, which have a pleated structure of β-strands linked by β-turns. Silk proteins feature less common helices with 3-fold symmetry, such as spirals with hydrogen bonding within (3_10_ helices) and between (3_1_ helices) protein strands.^20−22^ A protein’s secondary structural elements interact to fold into a tertiary structure, which can associate with other polypeptide chains to form higher order assemblies.

**Figure 1:**
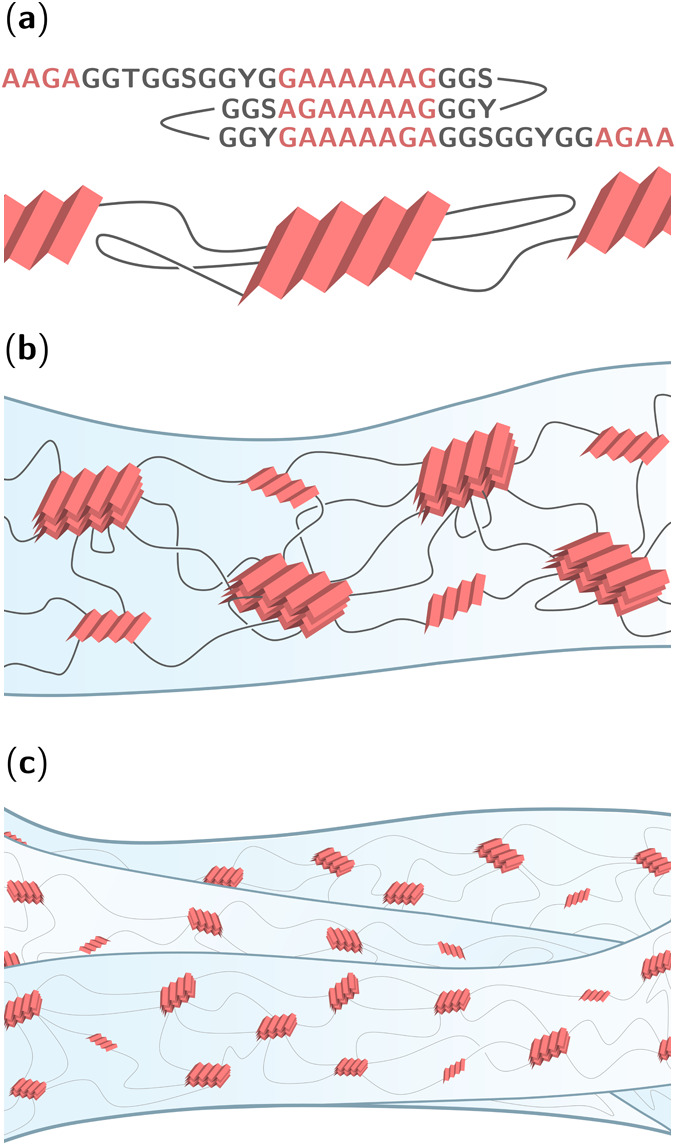
Hierarchical structure of silk fibers. (a) Illustration of a generic silk protein, where alanine-rich regions (red) form pleated β-sheet structures linked by flexible glycine-rich regions (gray). (b) Intramolecular and intermolecular β-sheets stack together into β-crystallites, which act as stiff nodes in a silk nanofibril’s “nanofishnet” structure. (c) Silk nanofibrils are twisted together to form microscopic silk fibers.

Silk from *B. mori* silkworms contains two fibroin fibers coated in a glue-like sericin protein. Silk fibroin is composed of a heavy chain (∼390 kDa), a light chain (∼26 kDa), and a small glycoprotein which maintains the integrity of the complex known as P25 (∼30 kDa).^23−25^ The heavy chain resembles a block copolymer with hydrophobic and hydrophilic regions and is primarily responsible for the mechanical properties of the cocoon fibers. It contains mostly alanine (A), glycine (G), tyrosine (Y) and serine (S), in GAGAGS, GAGAGY and GAAS motifs, the former constituting antiparallel β-sheets and the latter β-turns.^24,26,27^ In a silk nanofibril, dispersion forces and hydrophobic effects promote the packing of these β-sheets into strong “β-crystallite” nanocrystals linked by flexible regions,^28^ shown in Figure 1. In contrast to *B. mori* silkworms, a typical orb-weaving spider produces at least seven different spider fibroins,^29^ known as spidroins. Dragline silk, used as a lifeline and as radial web threads, is the most studied type of silk fiber due to its mechanical properties.^4^ Dragline silk is a combination of proteins from the spider’s major and minor ampullate glands, most of its properties arising from two major ampullate spidroins, MaSp1 and MaSp2. These proteins consist of a highly repetitive core region, with alanine-rich and glycine-rich sections, flanked by small N- and C-terminal domains.^30^ The alanine-rich regions form strong β-sheets, while the glycine-rich domains impart high toughness and extensibility.^31^ In MaSp1, these glycine-rich regions consist of GGX motifs in 3_1_ helices,^32−35^ whereas MaSp2 is also rich in proline (P), with GPGGX motifs that constitute type II β-turns and convey high elasticity.^36−39^

Silks from *Nephila clavipes* spiders, which have striking golden orb webs due to the presence of carotenoids,^40^ are particularly well-studied.^34,41−55^ Silks from other members of *Nephila* as well as the European garden spider, *Araneus diadematus*, have also often been investigated.^56−60^ Properties of silk fibers can vary substantially between spider species, due to the emergence of amino acid motifs as spiders adapted to their environments, changing fiber structure and the spinning process accordingly.^61,62^ Natural selection can also explain the superior mechanics of spider dragline fibers compared to silkworm cocoon silks: silkworms benefit from robust cocoons that prevent propagation of damage,^63^ while major ampullate spider silks must be tough, strong, extensible, and adaptable to environmental changes for optimal prey capture.

### 1.2 Rationale for Modeling Silks

Millions of years of evolution have refined the properties of silks at all length scales, as mutations in DNA altered the primary amino acid sequence of fibroins, thereby changing silk fibers’ mechanical properties from the bottom up. A comprehensive understanding of these fibers must therefore be reached from investigation at multiple length scales, facilitated by computational modeling which can correlate atomistic changes with higher order structure and properties. The approximately biannual doubling of computational power (as predicted by Moore’s law^64^) has made *in silico* modeling a far more powerful tool over the last few decades. Modeling can provide theoretical explanations of experimental results, enable investigation of systems not easily studied in the lab and facilitate the rational design of novel materials.

Here, we discuss how the field of modeling silk-based materials has developed as computational capability has improved, drawing particular attention to developments since 2019,^65^ such as large scale atomistic simulations of silk nanofibrils and the application of machine learning.^66−71^ We highlight the potential, and limitations, of using computational techniques to study natural silk fibers and novel silk-based materials.

This review begins by introducing common methods that have been used to model silk, from atomistic to continuum scales. We then discuss the applications of modeling to natural silks in understanding their structure, self-assembly and mechanical properties, as well as the response to varied environmental conditions of silk fibers and spider webs. Some recent applications of modeling to the design of synthetic silk-based and silk-inspired materials are then featured, before we conclude with speculation on the future of multiscale modeling of silk-based materials.

## 2 Methods for Modeling Silks

In computational research, a compromise must be made between simulation accuracy and computational cost.

*Ab initio* methods like density-functional theory (DFT) can predict highly accurate energies, structures, and spectroscopic data like nuclear magnetic resonance (NMR) shielding tensors, vibrational frequencies and optical absorbances. However, while quantum mechanical methods can be highly accurate, steep scaling with respect to system size makes them too computationally expensive to use at large length scales, such as modeling silk proteins which have thousands of atoms.

As a result of the infeasibility of applying DFT to large molecules, and the assumption that quantum effects are insignificant in these systems, classical approaches based on molecular mechanics have most often been used for atomistic modeling of silks. In these methods, the potential energy between atoms, *U*, is calculated as a sum of contributions from bonded and nonbonded interactions,^72^ by
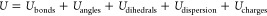


These potential energy functions are usually parametrized to fit quantum mechanical data and define a “force field” used to determine the forces on the atoms. These forces can be used to update atomic positions repeatedly until the forces vanish, at which point the structure has been optimized to a local energy minimum. Commonly used force fields to study silks have been AMBER,^73^ CHARMM,^74,75^ OPLS,^76^ and PCFF.^77^ Water molecules have often been described explicitly with the TIP3P model,^78^ although sometimes implicit solvation models are used to allow investigation over longer time scales.^79^

**Figure 2:**
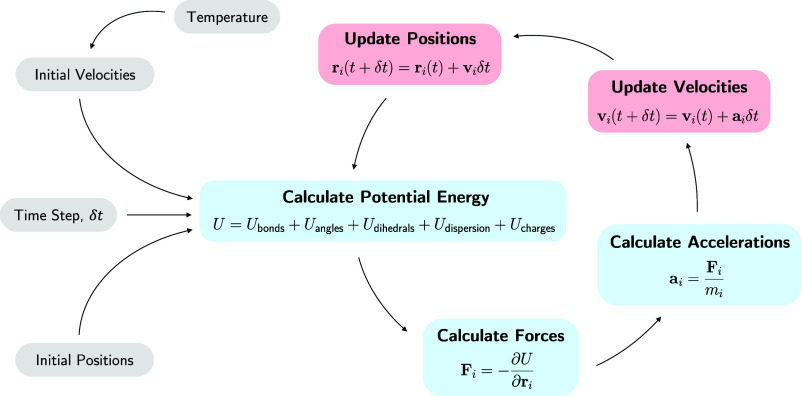
Simplified algorithm for the calculation of a trajectory in molecular dynamics. Calculations require an initial set of atomic positions and velocities, the latter set by the temperature, and an integration time step, *δt*, which is usually on the order of femtoseconds for atomistic studies. Potential energy, *U*, is calculated as a sum of bonded (covalent bonds, angles and dihedrals) and nonbonded (dispersion forces and electrostatic interactions) contributions, and the force, **F**, on the *i*th particle is then determined for all atoms. These forces are used to calculate accelerations, **a**, based on the particle’s mass, *m*, before velocities, **v**, and positions, **r**, are integrated by algorithms such as shown in the pink boxes. Potential energy is calculated again from these new positions, and time is moved forward by *δt*, continuing the cycle and mapping out a trajectory.

Molecular dynamics (MD) uses the force field to calculate particles’ accelerations by Newton’s laws of motion. The initial distribution of velocities is determined by the temperature, before these velocities and particle positions are updated in the calculation of a trajectory as shown in Figure 2. To study silk proteins, trajectories have often been calculated in the isothermal–isobaric thermodynamic ensemble, where temperature and pressure are held constant by algorithmic thermostats and barostats.^80−84^ Quantum and classical methods can also be combined in *ab initio* MD,^85^ which has been used to investigate the diffusion of water through a silk fibroin crystal, using DFT-calculated forces to update atomic positions by the classical equations of motion.^86^

Simulations with MD often suffer from the system becoming trapped in a local, rather than global, minimum on the free energy surface. The free energy landscape can be explored by increasing the length of the simulation, but this can be computationally infeasible, with atomistic time scales normally limited to a few hundred nanoseconds.^65,72^ To counteract this, several methods to enhance sampling have been developed.

One such method is umbrella sampling, where several simulations are run at different points over a variable of interest, such as a distance between two molecules, before the simulations are stitched together to give free energy as a function of that coordinate.^87^ Another widely used method to study proteins is replica-exchange molecular dynamics (REMD), where configurations of a system at different temperatures are periodically swapped, allowing free energy barriers to be overcome.^88^ A more recently developed method is well-tempered metadynamics, where a history-dependent potential is applied to the system that forces it to rise out of local minima.^89^ In a study of spider silk N-terminus dimers, conventional MD suggested that NaCl had no effect on salt bridges between the proteins. However, a metadynamics simulation revealed that this was an artifact of poor sampling — the state with broken bridges was more stable, but the original simulation trapped the proteins in a metastable local minimum.^90^

These enhanced sampling methods allow study of a protein around its equilibrium structure, but to investigate nonequilibrium systems, steered molecular dynamics (SMD) simulations are performed. These impose artificial forces on a system, and have often been used to generate stress–strain curves for silk proteins.^71,90−95^

Force field methods can also be used with coarse-graining techniques such as dissipative particle dynamics,^96^ where groups of atoms are replaced by single entities to reduce computational cost. This allows exploration of longer time and length scales at the cost of atomistic detail. A coarse-grained force field has recently been parametrized for spider silk, based on atomistic data, which should provide a reliable framework for investigating silks at larger scales in the future.^97^ At the largest length scales, finite element simulations are used. Here, the differential equations governing the system of interest are split into discrete points and solved numerically. Parameters for these higher scale calculations may come from experiment or a smaller scale MD simulation. Computational fluid dynamics simulations, where the Navier–Stokes equations that govern fluid flow are solved numerically,^98^ have also been used to study silk-based materials. For instance, silk nets from aquatic caddisfly larvae on the bottom of streams were shown to slow down water flow by up to 60%, creating a low-flow refuge for other organisms.^99^

A recent development in protein modeling has been the application of machine learning. In these models, algorithms are trained on a data set containing reference input and output data. The model’s predictions are compared to the true output values and the model adjusts itself to improve. This process is iterated over the full training set of data, improving the model’s predictive accuracy as it “learns” correlations between input and output data.^67,100^ A widely used machine learning model that predicts protein structure from amino acid sequence, using evolutionary data from sequence alignments, is AlphaFold.^70,101,102^ Other machine learning models have recently been developed to predict secondary structure content from amino acid sequence,^69^ and, conversely, to predict sequences from secondary-structural constraints to assist in material design.^103^

**Figure 3:**
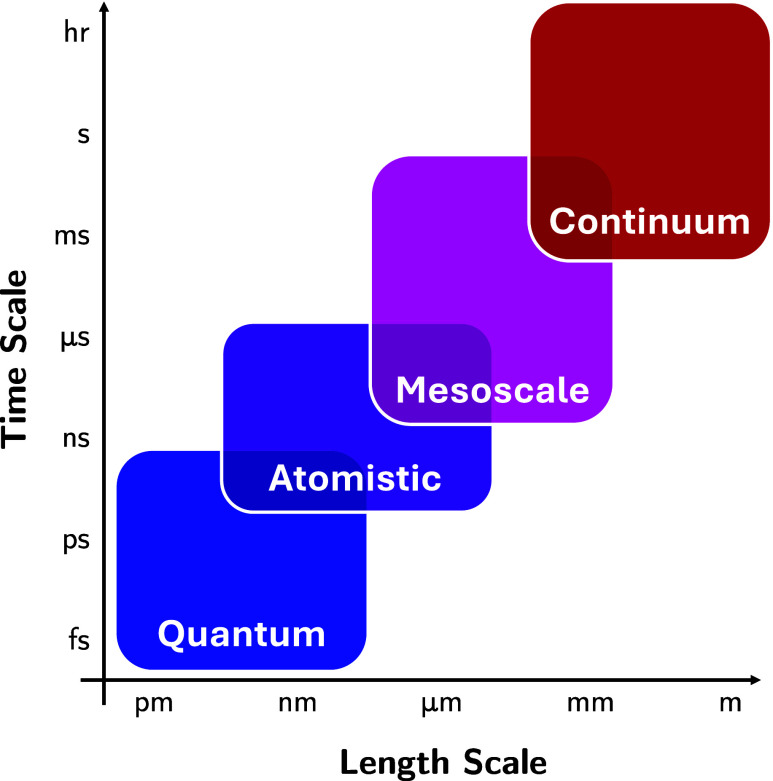
Characteristic time and length scales to which computational modeling methods are applied. At the smallest length and time scales, quantum mechanical methods such as DFT can be used. Beyond the nanometer and nanosecond scales, methods based on molecular mechanics are often employed, such as atomistic MD or coarse-grained simulation methods like dissipative particle dynamics at the mesoscale. At macroscopic lengths and long time scales, continuum methods such as finite element analysis and computational fluid dynamics are normally used.

Modeling across multiple length and time scales by the methods shown in Figure 3 can provide a holistic understanding of a material, linking fundamental molecular structure to emergent properties.^100^ Due to advances in computer power and software ease of use, performing computational simulations has become more straightforward in recent years. However, effective use of these tools to reach confident conclusions requires careful consideration of both the structural model and the simulation method, to balance accuracy and computational cost. Moreover, it is important that modeling studies be reproducible and verified by comparison to a finer-grained simulation and ideally to experimental data.^104^ But when applied effectively, multiscale modeling can be a powerful predictive tool and provide valuable insights into mechanisms that are challenging to investigate experimentally.

## 3 Natural Silk Fibers

Silk-producing animals serve as inspiration for materials scientists and engineers, helping us develop strategies for the fabrication of high-performance functional materials. Consequently, it is important to understand how the structure of silk fibers relates to their exceptional properties, and how this hierarchical structure assembles during the spinning process *in vivo*.

### 3.1 Structure Determination

Modeling has helped determine silk structures for a variety of organisms, for instance supporting predictions from bioinformatics that bee and wasp silks have an α-helical coiled-coil structure,^105−107^ but the main subjects of investigation have been silks from silkworms and spiders. Many early computational studies on silks focused on refining structural models, often by calculating spectroscopic data for model systems and comparing to experimental spectra.

Silk from *B. mori* forms different solid-state structures depending on its environment. The structure formed in the middle silk gland before spinning is silk I,^108^ and contrasts the structure formed after spinning, silk II, and the more recently discovered silk III, which forms at the air–water interface of regenerated silk fibroin solutions.^109,110^ Many applications of DFT to silks have been in the field of NMR crystallography, where data from quantum mechanical calculations, diffraction and NMR are used to refine crystal structures.^111−113^ For example, an early study calculated shielding tensors for silk I and II and compared to experimental NMR spectra.^114^ Limited computational power meant that only a short five-residue peptide was modeled, but the shielding tensors were demonstrated to be much more sensitive to the dihedral angles, ψ and φ, than to the long-range structure. While silk II was found to have antiparallel β-sheets, silk I was poorly described by any of the most common secondary structural motifs (α-helices, β-sheets and 3_10_ helices) in agreement with a study comparing calculated and experimental diffraction patterns.^115^ Later MD simulations and NMR experiments found that silk I consists of type II β-turns,^116^ the presence of which remains the consensus today. These turns are found in highly ordered regions of silk I known as silk I*, while the remaining sections of silk I are randomly coiled.^108^ The antiparallel β-sheet structure of silk II has recently been clarified based on data from dipolar-assisted rotational resonance spectroscopy, as a stack of lamellar structures in which adjacent protein strands have alanine methyl groups pointing in opposite directions.^117,118^

A recent DFT investigation studied decapeptide models of *B. mori* silk. Alanine was shown to retain the pleated β-sheet structure even in a single β-strand, avoiding alternative conformations that would interfere with stacking into a β-crystallite.^119^ These β-crystallites impart high strength to silkworm cocoons, so efficient packing enabled by alanine residues would be strongly favored by natural selection. Stabilization energies for interactions between oligopeptide chains were also calculated to investigate the strength of β-sheets, finding that oligo(glycine–serine) peptides were more stabilized than oligo(glycine–alanine) peptides. However, these stabilization energies differed by only a few kcal mol^–1^ so should be treated with caution, as thermodynamic differences on this scale are not reliably calculated even by highly accurate quantum chemical methods.^120^

Modeling was instrumental to an early study on *N. clavipes* dragline silk, where calculated solid-state NMR powder patterns were compared to experimental spectra to determine the orientation of alanine residues with respect to the fiber axis.^122^ Alanine’s methyl groups were shown to be preferentially aligned at 90^*◦*^ to the axis, but a less oriented region of alanine residues was also found to exist. These residues make up intramolecular, unstacked β-sheets (such as shown in Figure 1), constituting a semicrystalline region of spider silks, but not silkworm silks.^123^

**Figure 4:**
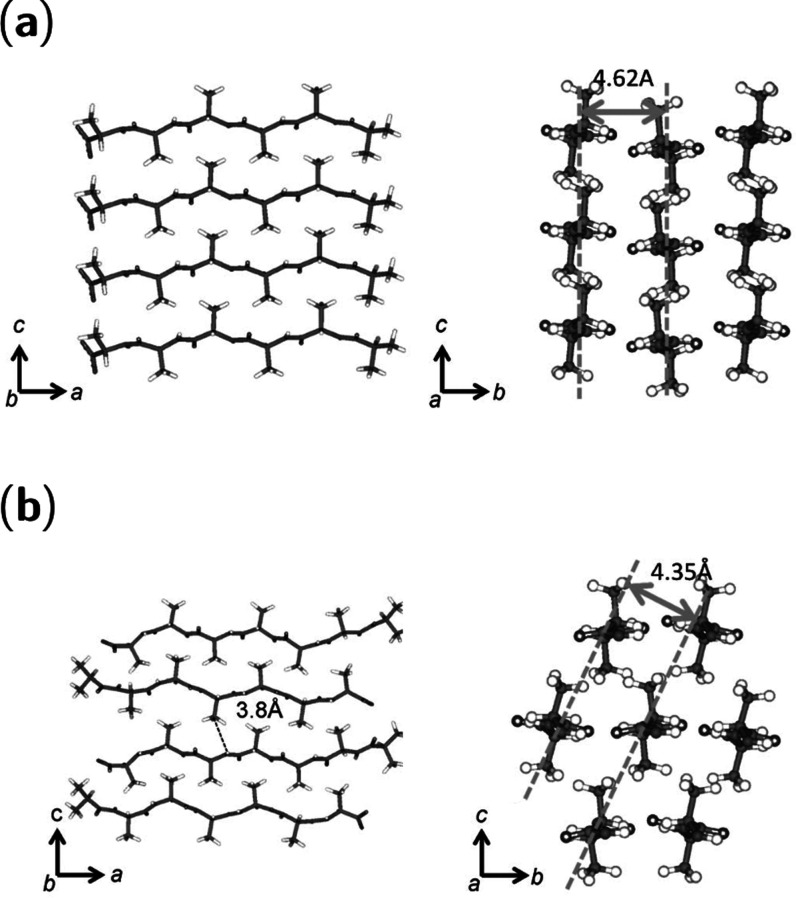
Structural comparison of oligo(alanine) β-crystallite structures (antiparallel short form and long form of polyA). (a) Rectangular structure of β-crystallites formed by A_6_ oligopeptides. (b) Staggered β-crystallite structure formed by A_7_ oligopeptides. Reproduced with permission from ref (121). Copyright 2012 John Wiley and Sons.

More recently, spider silks have been studied with atomistic MD. Studies on black widow (*Lactrodectus hesperus*) dragline silk revealed ring-packing CH−π interactions between proline and tyrosine residues and the presence of cystine slipknot motifs, consisting of three intertwined disulfide bonds, in low molecular weight cysteine-rich proteins present in the fiber.^38,124,125^ Many structural investigations of spider silks have used model peptides to correlate data from atomistic simulations and solid-state NMR.^52,121,126,127^ For instance, oligo(alanine) models were used to investigate packing in the stiff regions of *N. clavipes* silk.^121^ Packing between the antiparallel β-sheets formed was shown to be rectangular for small β-crystallites, but exhibited a staggered structure in larger nanocrystals, shown in Figure 4. It has since been demonstrated that the packing structure is dependent on the solvent with which the peptides were insolubilized from aqueous solution, with atomistic simulations showing that the rectangular-to-staggered transition could be induced by heat treatment, removing bound water.^52,127^

Spider silk’s glycine-rich region is often described in the literature as “amorphous”, but its true structure is a matter of debate. There is ample spectroscopic evidence that 3_1_ helices, with 3-fold symmetry and interstrand hydrogen bonds,^22^ are present in MaSp1, oriented parallel to the fiber axis.^32−34,128^ However, atomistic simulations have often failed to confidently predict these secondary structural elements, instead predicting random coils, β-turns and 3_10_ helices,^46,55,129^ which have 3-fold symmetry but form intrastrand hydrogen bonds.^21^ Since secondary protein structure is highly dependent on hydrogen bonding, force fields that do not sufficiently consider the directionality of these bonds due to covalency may be to blame.^20,130^ An alternative explanation has recently been proposed; atomistic simulations showed that the population of 3_1_ helices increases substantially upon changing the solvent from water to octanol, suggesting these helices may form late in the spinning process, stabilized by the low dielectric environment of the fiber.^35^ However, the existence of 3_1_ helices in dragline silk has been challenged, as a combination of antiparallel β-sheets, β-turns and random coils was shown to agree well with experimental solid-state NMR results on model peptides.^46^ This suggests the atomistic simulations may have been reliable, demonstrating the utility of modeling for cross-validating experimental results.

### 3.2 Self-Assembly

Understanding mechanisms involved in the natural formation of silk fibers has inspired synthetic processing techniques, such as acidification, application of shear stress, and macromolecular crowding.^18,131−134^ Moreover, an understanding of the formation of β-sheet-rich structures may have implications for Alzheimer’s and Parkinson’s disease, where such aggregates are pathological.^135^*In silico* modeling has provided insight into *in vivo* self-assembly, such as recent atomistic simulations highlighting the role of the less common amino acids tyrosine and arginine.

Comparing experimental and theoretical electron paramagnetic resonance spectra showed that a stable tyrosyl radical exists in *B. mori* silk’s hydrophobic regions.^136^ These radicals are thought to pair up to form dityrosine bonds, found in silks of several spider species.^41^ Atomistic MD has shown that pairing between tyrosine residues occurs spontaneously in solution, due to π–π and OH−π interactions, and templates β-sheet formation by reducing local motion and inducing hydrogen bond formation.^137^ Tyrosine’s importance has since been tested, as MD simulations on *Euprosthenops australis* MaSp1 mutants where tyrosine replaced other polar residues in GGX repeats showed that stronger dispersion and electrostatic interactions due to the presence of tyrosine increased dimer formation.^138^ These designed mutant proteins were then expressed recombinantly in *Escherichia coli* bacteria, and shown to self-assemble into nanofibrils with higher stiffness and β-sheet content than those composed of wild-type proteins.

**Figure 5:**
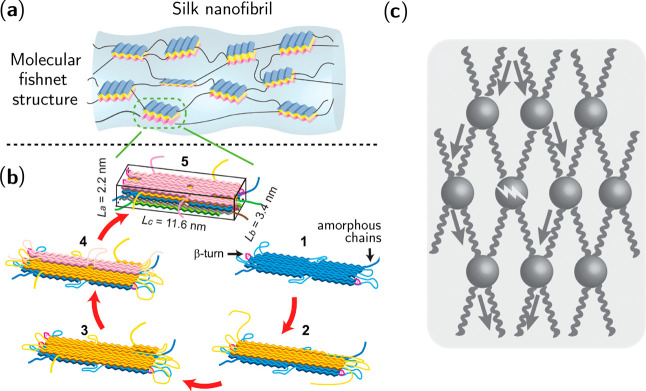
Nanofishnet structure of silk nanofibrils. (a) Schematic of stiff β-crystallite nodes joined by flexible linkers in a nanofishnet structure. (b) Formation of a large β-crystallite (such as found in *B. mori* silk) by stacking of β-sheets due to dispersion forces and hydrophobic effects. (c) Illustration of how the nanofishnet structure lets stress, shown by the gray arrows, bypass a damaged node, imparting the fiber with high strength and toughness. Adapted with permission from ref (28). Copyright 2016 John Wiley and Sons.

Figure 5 illustrates the formation of one of the key factors responsible for silk fibers’ mechanical properties, their nanofishnet structure. Here, stiff β-crystallites are joined by flexible linkers, enabling stress to bypass a damaged node thereby providing fibers with high strength and toughness.^28^ The spontaneous formation of this fishnet has recently been demonstrated by atomistic simulations, and shown to be dependent on humidity and the density of arginine residues.^139^ In excess humidity, β-sheets aggregated together, but this could be prevented by increasing the proportion of arginine residues in silk’s glycine-rich region, leading to nanofishnet formation and improved mechanical properties. Conversely, in adequate humidity, point mutation of arginine led to aggregation of fibroin molecules. The increase in toughness from the nanofishnet, at 15%, was much less than the 80% improvement suggested previously,^28^ likely due to the short length of the modeled fibroins — a longer fiber with more nodes will benefit more from the sharing of stress.^139^

#### 3.2.1 Influences from pH and Ions

The transition of silkworm and spider silk proteins from random coil to highly oriented fiber is facilitated by a pH gradient and interactions with various ions.^4^ At the start of the spinning duct, pH is neutral and there is a large concentration of Na^+^ and Cl^–^ ions, but along the spinning duct, these ions are replaced by an acidified solution of K^+^ and PO_4_^3–^ ions.^51^

The acidification-induced dimerization of fibroin N-termini is thought to be a crucial early step in self-assembly,^140^ and has been the subject of atomistic MD simulations.^141^ When pH fell below 6, glutamate residues became protonated and therefore more hydrophobic, promoting dimerization. This evidence has since been corroborated by further simulations which showed strong dipole–dipole interactions between N-terminus monomers at pH 6,^142,143^ and bioinformatics studies that found several glutamate residues are well-conserved between spider species.^144,145^

The influence of Na^+^ and Cl^–^ ions, which are abundant in the stored form of silk fibroin, on the N-terminal domain has also been investigated. Well-tempered metadynamics simulations on *E. australis* N-termini showed that high concentrations of NaCl weaken intermolecular salt bridges between positively and negatively charged amino acids, inhibiting dimer formation and therefore preventing unwanted aggregation in storage.^90^ This was supported by SMD simulations, which showed that dimers fell apart more easily in high concentrations of NaCl. While NaCl seems to break salt bridges in *E. australis* N-termini, a recent atomistic study on a *B. mori* silk nanofibril model showed that Na^+^ ions formed their own ionic bridges between protein strands, improving the silk’s mechanical properties.^146^

Calcium ions are also thought to be important in self-assembly, promoting β-sheet formation in *B. mori* silk and strengthening caddisfly larvae silks by cross-linking phosphorylated serine residues.^92,147^ Simulations on the *B. mori* N-terminus with MD suggested that the chelation of Ca^2+^ ions by glutamate and aspartate residues induces the formation of α-helices that later transform into β-sheets.^148^ In the middle–posterior part of the *B. mori* silk gland, an average of 28 divalent calcium ions are present per chain of native silk fibroin,^149^ forming interstrand cross-links via negatively charged amino acids. A mathematical model quantitatively predicted the effect of breaking these links by increasing K^+^ concentration, leading to a decrease in silk’s viscosity.^150^ Coarse-grained simulations have since investigated these cross-links further, and the modulation of viscosity has been interpreted as a method to ease chain alignment under flow.^151^ However, downstream in the silk gland, higher acidity increases calcium bridge stability, thereby linking these elongated chains together, disrupting silk’s hydration shell and enabling efficient crystallization during liquid–solid phase separation.^152^

Similarly to the N-termini, spidroin C-termini dimerize during self-assembly.^153^ Upon acidification, the C-terminus undergoes a transition from an α-helical to a β-sheet-rich structure, driven by the self-assembly of a substituent helix which can form nanofibrils in the absence of the rest of the protein domain.^154^ However, the C-terminus has seen little computational investigation — future MD studies might demonstrate the mechanism of this self-assembly, and specifically the roles of conserved cysteine and leucine residues.^145^ Atomistic simulations might also investigate the interaction of silk with multiple metal ions as, to our knowledge, MD studies have only been performed with ions in isolation, in contrast to the complex composition of the dope in silk-spinning animals.

#### 3.2.2 Influences from Stress and Flow

Throughout the spinning process, silk proteins are subjected to large shear and longitudinal stresses, which promote the formation of strong and tough β-sheet-rich nanofibrils by enhancing first liquid–liquid, then liquid–solid phase separation.^44,152,155−157^

Early atomistic studies on model peptides of silkworm silk showed that β-sheet-rich structures were formed under high shear stresses, with the structural change accelerated by the presence of water molecules around the silk fibroin.^158,159^ More recent simulations have found that shear stresses on *N. clavipes* silk of 300–700 MPa are required for fiber formation,^160^ in agreement with the forces exerted in spiders.^161^ Testing the limits of shear stress, an MD study also investigated overshearing, which was found to promote a metastable semicrystalline state in highly extensible contracted fibers.^162^

Computational fluid dynamics has been employed to investigate the spinning process in silkworms, showing that increasing shear rate decreases viscosity up to a point, where shear-induced crystallization takes over and viscosity increases.^163−165^ The pressures required for fiber formation have been found to be higher than could be generated by extrusion from a silkworm. Natural spinning was therefore suggested to result primarily from the pulling of fibers by the silkworm, rather than from pushing of the feedstock.^166^ Fluid dynamics simulations have also been used to design a bioinspired microfluidic device based on natural silk spinning principles, producing fibers with 3-fold higher modulus and 12-fold higher toughness.^134^

The greater prestrain of major ampullate silk, resulting from high longitudinal stress during spinning,^56^ has been proposed to explain its high strength compared to minor ampullate silk.^168^ Recent advances in computational power have allowed large scale atomistic MD investigation into the effects of prestretching on silk fibroin’s mechanical properties.^71^ A model was developed by first equilibrating a block copolymer coarse-grained structure, before the amino acid sequence was threaded on to create the nanofishnet structure of a nanofibril with several β-crystallites. The prestretching process reduced β-sheet content but split up β-crystallites into smaller nodes well-aligned along the fiber axis. Strength increased from 800–1600 MPa after prestretching, while toughness decreased from 3500–1500 MJ m^–3^, correlating with mechanical data from threads spun with faster reeling speeds.^169,170^ Prestretching has also been investigated in the context of hydrodynamic flow, using a recently developed protocol to incorporate flow into MD simulations.^171,172^ A combined atomistic and coarse-grained study of *N. edulis* dragline silk showed that less extended proteins aggregated faster, but into less ordered assemblies, while proteins at larger extensions assembled reversibly into highly ordered structures.

#### 3.2.3 Models of Self-Assembly

Influences from stress and ions are clearly important in the self-assembly process, but do not by themselves provide a holistic understanding of the transition of silk proteins through the spinning gland. Fundamental models of the assembly of soluble fibroins into solid fibers have been proposed, the most well-known being the “liquid crystal” model proposed by Vollrath and Knight,^173^ and the “micellar” model proposed by Jin and Kaplan.^174^

In the liquid crystal model, silk fibroin molecules flow while maintaining orientational order, preventing unwanted aggregation but keeping the force required for fiber assembly low.^173^ Fibroin has similar numbers of hydrophilic and hydrophobic amino acids, so only small perturbations are needed for aggregation or solvation.^175^ The rod-like liquid crystals align in the same direction, forming an amphiphilic block copolymer before a liquid–solid phase transition aligns the β-sheet-rich structures together into a fiber.^173^

In contrast, the micellar model suggests that the repetitive regions of fibroin molecules constitute the interior of micelles, while the hydrophilic terminal domains are displayed on the exterior.^174^ The intervening hydrophilic sections of the repetitive regions remain hydrated, keeping fibroin in solution. When the concentration of fibroin increases, micelles merge into larger, globular structures before shear and longitudinal forces elongate these globules into solid fibers.

Of these two classic theories, recent results from modeling and experiment seem to favor the micellar model. Recombinantly produced silk-mimetic proteins experienced a phase transition from isotropic solution to an assembled structure.^176^ At low concentrations, small aggregates formed while at higher concentrations, spherical structures emerged. A coarse-grained bead–spring model was sufficient to explain this self-assembly. Moreover, the formation of fibrous structures from micelles has been demonstrated in an SMD study of a hydrogel model of prespun silk, in the presence of fast shear flow.^177^ Recently, analytical techniques have shown that silk fiber production *in vivo* involves the formation of microcompartments, reminiscent of the spherical structures predicted,^176^ and nanocompartments, resembling the smaller aggregates, which guide silk fibroin molecules into higher order structures in a reversible process.^178^ This highlights the potential for predictive power by even relatively simple computational models.

**Figure 6:**
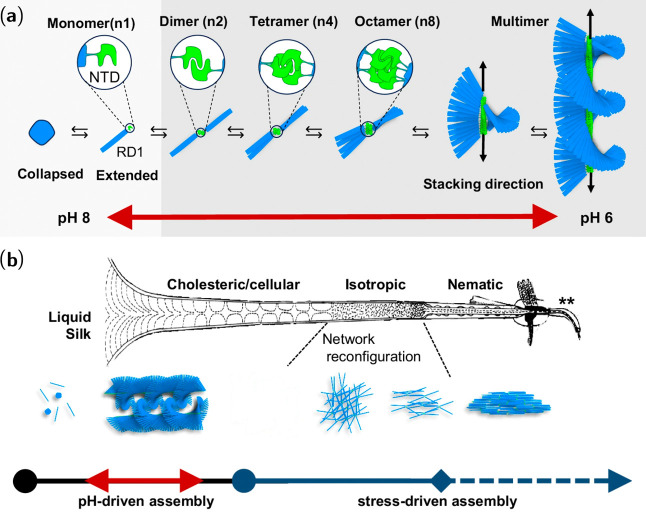
β-Solenoid structure and self-assembly of *B. mori* silk fibers. (a) Proposed pH-driven self-assembly of the β-solenoid, from globular monomer to multimeric bottlebrush-like fibril showing the proposed cholesteric order. (b) Summary of the suggested assembly pathway of silk fibroin, showing pH-driven assembly of the cholesteric phase, reconfiguration into a fractal network and stress-driven assembly into a nematic liquid crystalline phase and silk nanofibrils. Adapted with permission from ref (167). Copyright 2024 Springer Nature.

Though this evidence seems to support the micellar theory, it is important to note that the two common models are not mutually exclusive; low concentrations of lyotropic liquid crystals form micelles, but increasing concentration favors the formation of columnar structures.^179^ Reconciliation between these two models was recently proposed in a study combining spectroscopy and modeling, where the *B. mori* heavy chain was shown to exhibit a “β-solenoid” structure from oligomerization of N-terminal domains,^167^ as shown in Figure 6. The structure of silk I was predicted with AlphaFold2,^102^ and increasing acidity was suggested to induce stacking into the twisted, cholesteric β-solenoid, the structure of which could explain the experimental diffraction pattern of prespun fibroin. During self-assembly, these β-solenoids were proposed to be transported in globular microcompartments, before stress aligns the structure into a fractal network,^180^ and then a liquid crystalline nematic phase which can extend into nanofibrils.

### 3.3 Mechanical Properties

**Figure 7:**
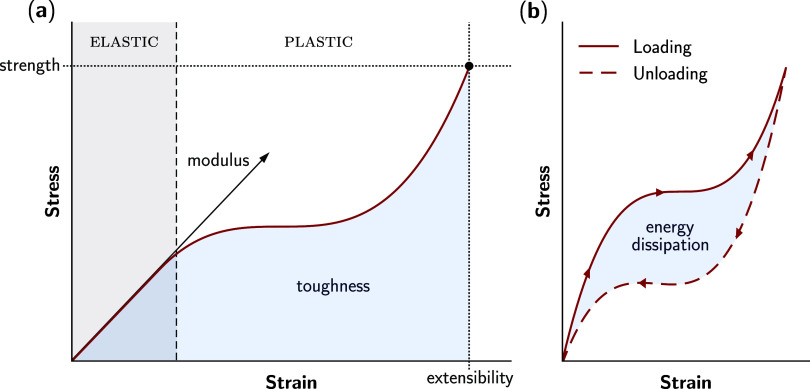
Anatomy of a stress–strain curve. (a) In the elastic region, stress is linearly related to strain, with the slope being the elastic modulus. Permanent deformation takes place in the plastic region, until the point of fracture, shown by the black circle. The strength and extensibility are the stress and strain at fracture respectively, and toughness is the area under the stress–strain curve. (b) The response of a viscoelastic material to cyclic loading, where the area difference between the loading and unloading curves encapsulates the energy loss due to internal friction. Hysteresis is the ratio of this dissipated energy to the energy absorbed.

Fiber mechanics are often described in terms of stress, which is force applied per unit area, and strain, the percentage elongation. The relationship between these variables can be visualized in a stress–strain curve, such as Figure 7.

These graphs are rich in mechanical information. Strength and extensibility are the stress and strain at fracture, respectively. Stiffness is the slope of the curve, and the area under the curve is the fiber’s toughness — the total capacity for energy absorbance. In the elastic region, stiffness is known as the elastic modulus, and deformation is fully recoverable, whereas deformation in the plastic region is permanent. Viscoelastic materials such as silk exhibit stress–strain hysteresis, where loading behavior differs from unloading behavior due to energy dissipation from internal friction.^53^

To the best of our knowledge, the first model which could rationalize the mechanical properties of silk fibers was reported by Termonia.^181^ Dragline silk was represented as a two-dimensional lattice of stiff nodes linked by rubber-like flexible regions. Although a simple model, this lattice reproduced several characteristics of silk’s stress–strain curve and correctly predicted that tensile strength would decrease as crystallite size increased.^182^ Since then, several further theoretical models have been developed to study different aspects of fiber mechanics.^183−192^ For instance, models have predicted silk fibers’ relaxation modulus,^183^ cyclic loading behavior,^185^ and explained the collapse of β-crystallites under stress.^186^

Relatively simple models have shown good agreement with stress–strain mechanics at the fiber scale. For example, early studies found silk’s strength could be quantitatively predicted by the degree of order in silk’s structure using mean field theory for polymers,^187^ and a model with no empirical parameters, using data from MD simulations, was able to predict fiber strength, toughness and elastic modulus.^188^ More recently, a “tensegrity” finite element model, composed of β-crystallite nodes under compression linked by tendons under tension, was able to reproduce stress–strain curves and show that the high toughness of spider silk fibers could be understood by radial variability in the ductility of these tensegrity units.^190^

Theoretical models have shown how fiber mechanics can be understood in terms of engineering principles, but atomistic simulations are necessary to link silks’ properties to their fundamental chemistry. A notable contribution to the field of modeling silks came from Keten and Buehler, who used REMD to develop an atomistic model for *N. clavipes* silk.^193^ This was able to predict Ramachandran plots^194^ and has inspired several subsequent atomistic studies of silk proteins.^45,54,55^ Machine learning may prove to be another key advance in modeling silks. Recently, data from SMD simulations has been used in machine learning models to predict the mechanical properties of silk fibers from their amino acid sequences,^66^ and to generate amino acid sequences that would produce proteins with desired mechanical properties.^195^ The small size of the data sets used limits the generality of these models, but since the data comes from SMD simulations, advances in computational power and algorithm efficiency promise to ease this limitation.

#### 3.3.1 Strength, Toughness and Stiffness

The mechanical properties of silk fibers fundamentally result from hydrogen bonding in antiparallel intermolecular and intramolecular β-sheets.^196^ These bonds are very weak individually, keeping self-assembly reversible,^178^ but when aligned together they can resist remarkable stresses, surpassing the strengths of man-made materials.^179^ Computational modeling has revealed how the arrangement of hydrogen bonds, rather than their strength, conveys exceptional mechanical properties to certain silk fibers.

Silkworm fibers have larger β-crystallites than spider silk fibers, but this does not translate to better mechanical properties — in fact, reducing β-sheet content has been shown to *improve* fiber mechanics.^197^ Spider β-crystallites are more highly oriented and smaller, allowing stress to spread more efficiently over nodes, making fibers tougher, stronger and less brittle.^28,91,182^ A multiscale model integrating MD and finite element analysis predicted fibers are toughest with a low crystallinity of 10–40%,^198^ such as found in spider silks. This low crystallinity also means that not all intramolecular β-sheets in spider silks are stacked into β-crystallites. These unstacked intramolecular β-sheets provide silk with its high initial modulus, and give rise to a nonlinear strain-stiffening response when hydrogen bonds reform between protein strands at high strains.^199,200^ One of the great successes of atomistic modeling of silk has been the development of the theory of “nanoconfinement”, which explains how limiting the size of β-sheets, β-crystallites and nanofibrils gives spider dragline silk extreme strength and toughness simultaneously.^201−203^

Long β-strands are very rarely found in proteins.^204^ Keten and Buehler proposed an explanation for this, showing that when many hydrogen bonds are placed under stress in a two-dimensional β-sheet model, only 3–4 break concurrently.^205^ Therefore, it was suggested that no additional strength benefit is provided by long β-strands. A subsequent study, with a series of large scale MD simulations, revealed that smaller β-crystallites (2–5 nm long), as found in spider silks, yield higher stiffness, strength and toughness compared to larger β-crystallites, such as found in *B. mori* silk.^201^ The hydrogen bonds in small nanocrystals failed and reformed in a concerted “stick–slip” motion, enhancing the dissipation of energy, whereas a crack-like flaw concentrated stress in larger crystals. The principle of geometric confinement was also shown by modeling to hold at the nanofibril level — narrower fibrils allowed silk fibers to reach higher ultimate stresses and strains.^206^ The optimal nanofibril diameter was predicted to be 50 ± 30 nm, in line with experimental measurements (20–150 nm).

Interestingly, while strength has been shown to plateau around 10 nm in length for isolated nanocrystals, a recent MD study found that strength increases for “ultra-long” β-crystallites, as hydrogen bonds work cooperatively to move these microcracks down through the nanocrystal, breaking in front of the crack while reforming behind it.^207^ The stick–slip regeneration of hydrogen bonds was also recently shown to explain the higher toughness of glycine-rich dragline silks compared to serine-rich eggcase silks, which have more hydrogen bonds but less regeneration during plastic deformation.^31^

The gain in mechanical performance per alanine residue in a β-sheet has been found to be maximal at eight residues,^208,209^ double the ideal number of hydrogen bonds found previously^205^ due to the more recent study using a three-dimensional β-crystallite model instead of a two-dimensional β-sheet. A subsequent study on a model of *N. clavipes* MaSp1 found that a minimum of six alanine residues is necessary for the formation of stable nanocrystals during spinning.^55^ These studies predict β-sheet-forming motifs in agreement with natural systems, as oligo(alanine) sections in spiders are either six or eight residues long, depending on species, suggesting spiders have evolved to be highly efficient with their resources. Moreover, atomistic MD simulations have suggested that this length scale optimizes the self-assembly of silk’s semicrystalline regions, imparting greater toughness.^210^

**Figure 8:**
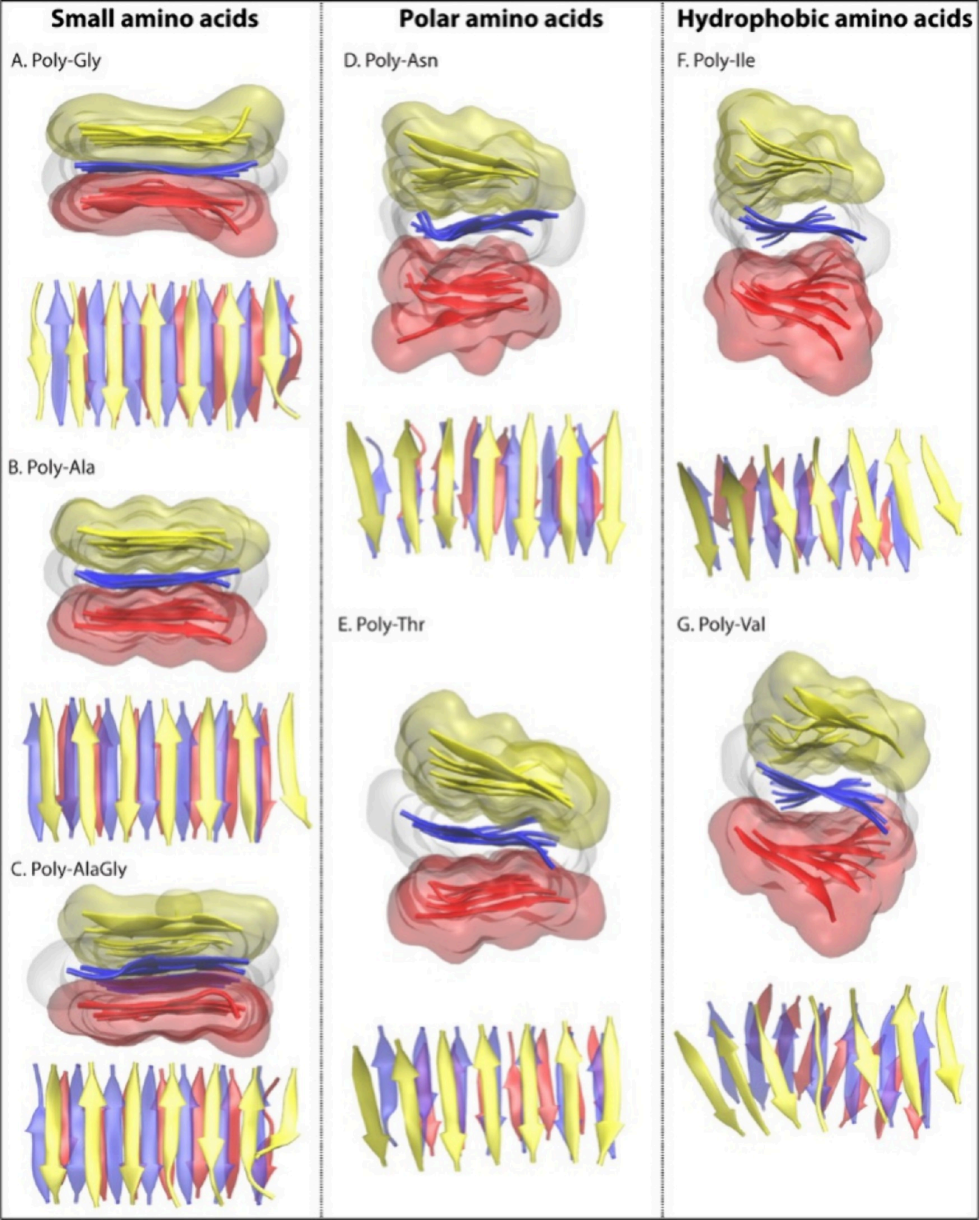
Shape and topology of homopolymer representative models. Figure shows side and top views of each homopolymer modeled nanocrystal post the equilibration step. Side view shows β-strands in the new cartoon representation with upper/bottom sheet depicted in quicksurf representation and ghost rendered quicksurf view of the middle sheet. In the top view of nanocrystal, β-strands are shown in the new cartoon representation as viewed from above the model. In each view, the upper, middle, and bottom β-sheet layers are shown in yellow, blue, and magenta red colors, respectively. Reproduced or adapted with permission from ref (94). Copyright 2021 American Chemical Society.

The extent of spider silk’s evolutionary refinement has recently been tested, by determining the mechanical performance of β-crystallites made from other amino acids than alanine, with SMD pull-out simulations.^94^ Several homopolymers of different amino acids (alanine, glycine, alanine–glycine, asparagine, threonine, isoleucine and valine) were used to form β-crystallites, but oligo(alanine) β-crystallites packed together the closest, as shown in Figure 8, and had the highest strength and toughness. It had previously been speculated that changing the β-crystallite composition could improve the strength of silks,^211^ but it seems that nature has optimized these structures for their ecological functions.

#### 3.3.2 Elasticity and Friction

Silk’s elasticity was the subject of an early MD study, where a β-sheet from *B. mori* was modeled as an elastic rod.^212^ Based on this assumption, silk fibroin’s elastic modulus was calculated, in reasonable agreement with experiment considering the simplicity of the model. Since then, elasticity has been the focus of several theoretical models,^189,191,192^ and atomistic modeling studies.^213,214^

The very high elasticity and extensibility of spider dragline silk has been proposed to result from the presence of proline in MaSp2, in type II β-turns which combine to form entropically recoiled β-spirals.^39,215^ This theory was recently supported by atomistic simulations on aqueous glycine-rich fragments of MaSp1 and MaSp2 in *Argiope aurantia* spider silk.^213^ Both fragments showed elastomeric behavior and shrunk dramatically from a stretched position, but the maximum recovery force was 60% higher in the proline-rich MaSp2 fragment compared to the MaSp1 peptide, correlating with the higher breaking strain in silks from species that have MaSp2 compared to those which lack the protein.^216^

The elasticity of silk is limited by internal friction in its flexible regions, which increases fiber strength by allowing protein chains to share stress.^198^ This internal friction is also thought to be the origin of silk’s viscosity and hysteresis.^53,57^

A REMD study on *N. clavipes* dragline silk demonstrated permanent changes in secondary structural composition after cyclic loading,^214^ as the ratio of random coils to β-turns did not return to its original equilibrium value, suggesting an avenue for energy dissipation. Atomistic MD later quantified the frictional forces in the flexible regions of *A. diadematus* silk,^57^ and on the flexible–crystalline boundary.^217^ The coefficient of viscosity was found to be 2 orders of magnitude lower on the boundary compared to within the flexible region, as the ordered β-sheet surface provides less hindrance than the tangled chains of the glycine-rich fraction. This low friction between silk’s soft and stiff blocks has been suggested to assist self-assembly, as it facilitates microphase separation and chain alignment under stress.^156^

While friction inside nanofibrils leads to hysteresis, friction between nanofibrillar bundles provides significant mechanical benefits. Dragline silk fibers have a twisted and globular morphology,^182^ which finite element simulations suggest allows frictional forces to restrict shearing and prevent crack propagation, while enabling local slipping under high stress to prevent bulk fracture.^182,218^ Additionally, coarse grained simulations showed that rougher silk fibers are tougher, as shear-locking between globules increased stress transfer by up to 200%.^219^ Mechanical benefits from apparent disorder in silks have also been shown below the fiber length scale, as randomly positioned MaSp1 bundles demonstrated higher ultimate strength under uniaxial stretching compared to fully ordered bundles in a study using a recently developed coarse-grained force field for silk.^97^ This force field, parametrized with atomistic simulation data, should provide a framework for the investigation of silk proteins at longer time and length scales than have been accurately accessible thus far.

#### 3.3.3 Dragline Silk’s Stress–Strain Curve

**Figure 9:**
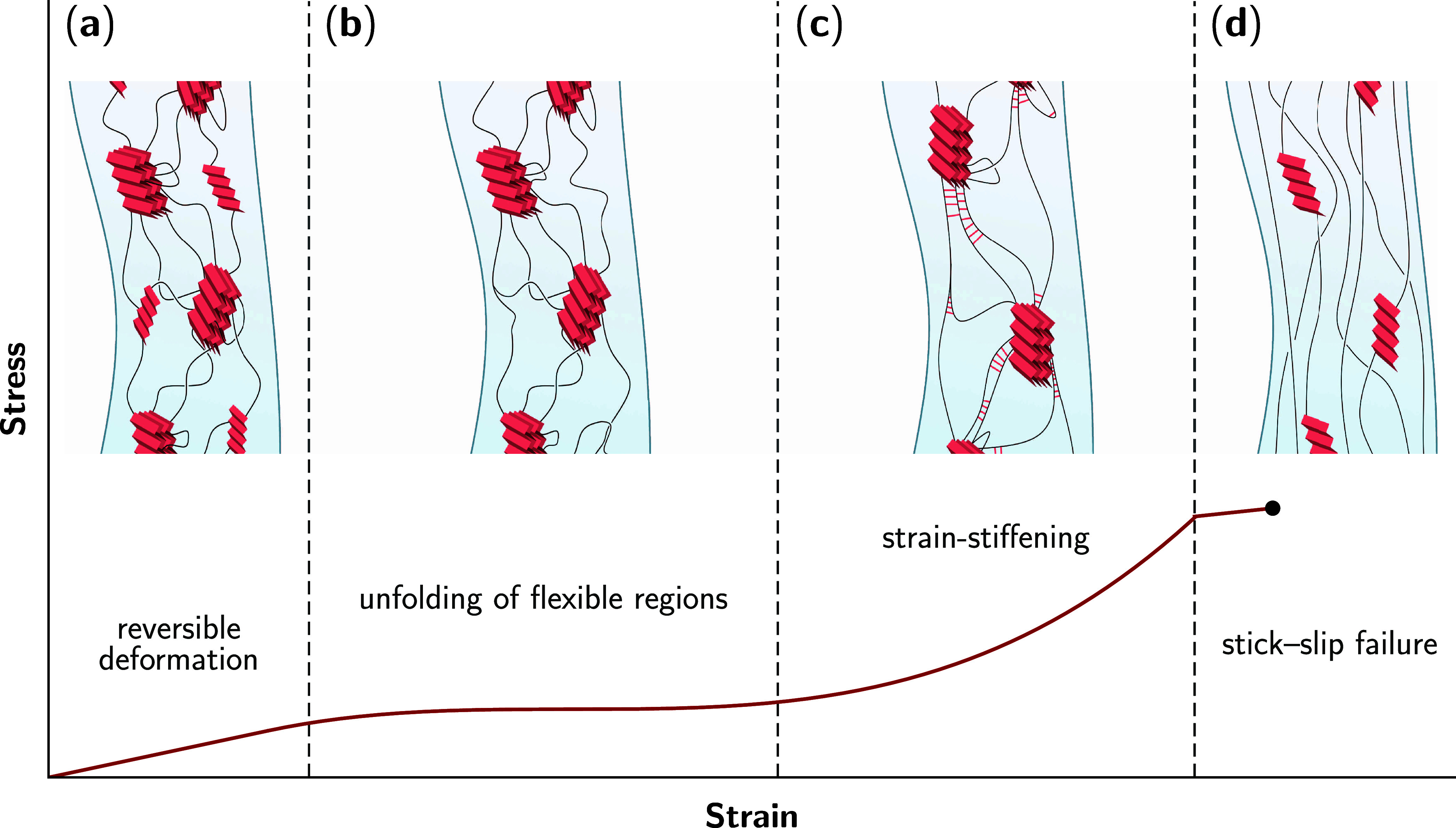
Molecular response underlying a dragline silk fiber’s stress–strain curve. (a) In the elastic region, hydrogen bonds in intramolecular β-sheets bend, able to reform upon release of stress. (b) After the silk fiber’s yield point, these β-sheets have been pulled apart and the entropically coiled flexible regions extend. (c) The flexible regions are close to fully extended and β-crystallites are well-aligned with the fiber axis. New hydrogen bonds (red lines) form between the stretched protein chains leading to strain-stiffening. (d) Finally, the hydrophobic and dispersion interactions holding β-sheets together in β-crystallites are overcome, as hydrogen bonds break in a stick–slip motion that causes a brief strain-softening before fiber failure, shown by the black circle.

Figure 9 summarizes the molecular mechanisms behind spider dragline silk’s response to applied uniaxial stress, elucidated through modeling and experiment.

The response of silk fibers to stress is initially elastic, with its high modulus resulting from the reversible distortion of intramolecular β-sheets in the semicrystalline region and intermolecular and intramolecular hydrogen bonds in the flexible, glycine-rich region.^20,93,184,200^ After these hydrogen bonds break, the fiber’s deformation becomes plastic and the entropically recoiled flexible regions extend. Hydrogen bonds between the intramolecular β-sheets and glycine-rich chains begin to reform, as β-crystallites split into smaller nodes which are highly aligned along the fiber axis, during strain-stiffening.^184,200,220,221^ Finally, the hydrophobic and dispersion interactions between β-sheets are overcome, as the β-crystallites collapse in a stick–slip motion, giving rise to a short strain-softening period immediately prior to failure when the fiber ruptures.^179,186,222,223^

### 3.4 Responses to the Environment

In developing silk-based materials it is essential to understand how fibers respond to different environmental conditions, such as changes in humidity and temperature. Understanding these responses, as well as the natural responses of spider silk fibers in their biological roles in webs, has therefore been a key target of modeling studies.

#### 3.4.1 Hydration and Supercontraction

Early atomistic simulations using single β-crystallite models found a clear decrease in *B. mori* silk strength with hydration, as water disrupted intermolecular hydrogen bonds.^224,225^ A recent atomistic study on a more comprehensive model, incorporating multiple β-crystallites linked by flexible regions, provided more nuanced results.^226^ As hydration increased, there was an initial increase in strength and elastic modulus due to augmentation of the hydrophobic effect, but at high humidity the disruption of hydrogen bonds became more significant, weakening the silk fiber. Similarly, recent MD simulations have shown that hysteresis initially increases with hydration, before the effect is diminished when high water levels plasticize silk chains.^227^

Dehydration makes spider silks brittle,^228^ but evidence from atomistic simulations suggests that spiders have evolved mechanisms to counteract this vulnerability.^229^ Models of wild-type *N. clavipes* dragline silk were compared to β-crystallites made of oligo(alanine) and oligo(alanine–glycine). The wild-type model exhibited the best mechanical properties under dry conditions, with a superior water collecting ability. Moreover, in high humidity, the artificial models, but not wild-type silk, saw a decrease in β-sheet content, suggesting that spider silk has evolved to be adaptable to varying levels of moisture.

An intriguing property of some spider silks is their ability to shrink by up to half their length in high humidity, a phenomenon known as “supercontraction”.^59^ Potential biological roles of this contraction have been proposed: a web that stiffens in the morning dew would reduce sagging from heavy water droplets, and let vibrational signals travel to the spider more quickly.^230,231^ These hydration-induced contractions are highly reversible and generate work 50 times greater than that from the equivalent mass of human muscle fibers,^10,232^ prompting interest into using spider silk in humidity sensors and artificial muscles.^10,11,233,234^

Supercontraction is thought to result from silk’s flexible chains recoiling into a higher entropy configuration after water disrupts intermolecular hydrogen bonds.^122,235,236^ Evidence from atomistic simulations and Raman spectroscopy has suggested that certain polar residues are vital for the effect.^235^ Upon hydration, tyrosine’s hydrogen bonding behavior was shown to change from mostly donating hydrogen bonds to mostly accepting them. Moreover, *in silico* point mutations of tyrosine and arginine led to elimination, and even reversal, of supercontraction.

**Figure 10:**
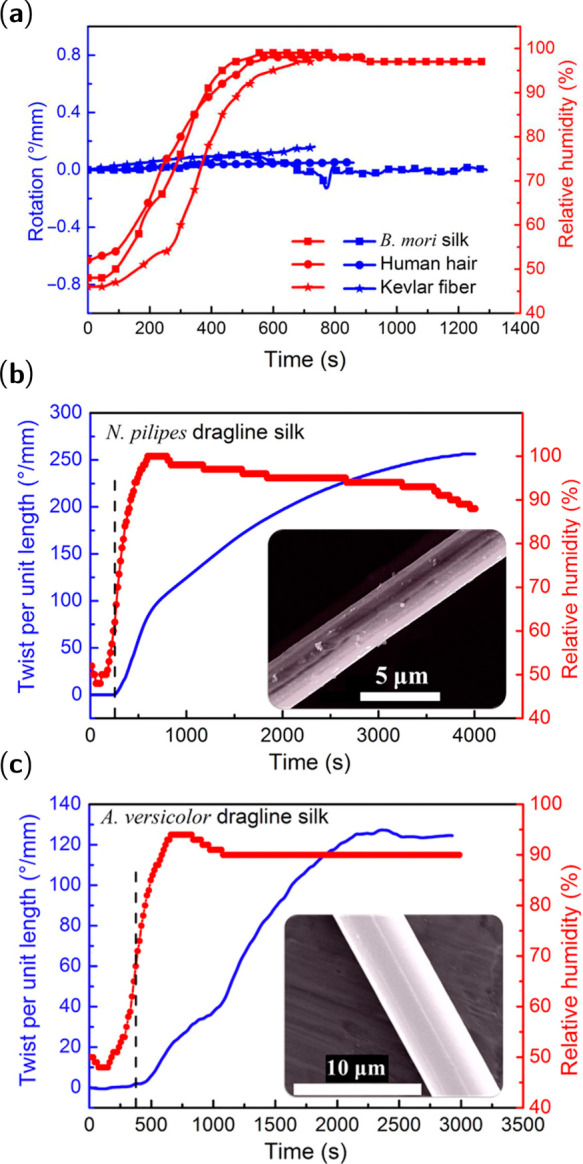
Torsional actuation of silks with increasing the relative humidity from 40 to 100%. (a) Torsional responses of the representative fibers to environmental humidity: B. mori silk fiber (65.1 mm in length), human hair (69.5 mm in length), and Kevlar fiber (86.9 mm in length). A negligible twist driven by humidity can be seen in these fibers. (b) Torsional actuation of *N. pilipes* spider dragline silk (121 mm in length, 3.1 ± 0.1 μm in diameter). (c) Torsional actuation of *A. versicolor* spider dragline silk (87.9 mm in length, 6.7 ± 0.1 μm in diameter). Inset shows the SEM images of representative silks. Reproduced with permission from ref (11). Copyright 2019 The American Association for the Advancement of Science.

These simulation results have since been further supported experimentally, as tyrosine residues were found to mediate supercontraction in biomimetically produced artificial silk fibers.^237^ Proline had previously been thought to be crucial, as supercontractile ability correlates with the proportion of proline residues in a fiber.^238^ However, supercontraction was still observed in fibers void of proline, but not in fibers void of tyrosine.^237^ In addition to supercontraction, spider silks have been found to undergo a twist in response to humidity, as shown by Figure 10. Atomistic MD simulations have linked the bulky ring-shaped structure of proline to this torsion, suggesting that steric exclusion disrupts hydrogen bonding in the presence of water and induces a twist in MaSp2.^10,11^

On a larger scale, an analytical model that predicts supercontractile stress as a function of relative humidity has been proposed, modeling silk as a block copolymer embedded in an elastic matrix.^236^ The model was found to be in good agreement with experiment with only one arbitrarily fitted parameter. This parameter quantifies the decrease in intermolecular hydrogen bonds above a humidity threshold — its value could perhaps be justified in a future atomistic study.

Recently, a novel machine learning technique has been developed, which allows the determination of best-fitting analytical expressions that link fundamental and emergent properties of hierarchical materials.^68^ Silk’s macroscale properties, like fiber diameter and stress–strain mechanics, were correlated with mesoscale and microscale properties, such as birefringence, thermal stability and primary protein structure. Supercontraction could not be reliably predicted from the mesoscale properties but was predicted surprisingly well from microscale properties directly. A simple expression involving the length of the repetitive region of MaSp2 and the length of the oligo(alanine) motif in MaSp1 could predict fiber scale supercontractile ability, suggesting a possible direct link between atomistic and macroscopic properties.

#### 3.4.2 Thermal Conductivity

Dragline silk from *N. clavipes* was reported to have exceptionally high thermal conductivity, around 400 W m^–1^ K^–1^, comparable to that of copper.^239^ Atomistic simulations were later performed to understand the origin of this newly discovered property.^240^ Stiff interstrand hydrogen bonds in β-sheets prevented the loss of phonon coherence and blue-shifted low frequency vibrational modes, leading to much higher conductivity than other proteins tested in the same way. However, the calculated thermal conductivity was around 4 W m^–1^ K^–1^, orders of magnitude below previously reported experimental results, and the calculation is likely an overestimate due to neglect of intersheet interactions and silk’s flexible regions.^240^

This hundredfold discrepancy seemed to suggest serious limitations in the calculations, however the original experimental study^239^ has since been refuted,^48,241,242^ as heat loss from silk fibers due to radiation was not considered. More recent experimental estimates of spider silk’s thermal conductivity predict a value of 1.2 W m^–1^ K^–1^,^48^ much closer to the simulation results. This highlights how computational modeling can have genuine predictive power — a significant disagreement between simulation and experiment means data and methodology should be analyzed carefully to understand the origin of the discrepancy.

*B. mori* silk, with thermal conductivity of 0.8 W m^–1^ K^–1^, is somewhat less susceptible to phonon propagation than spider silks despite its higher crystallinity.^242^ To explain the difference, nonequilibrium MD simulations investigated atomistic models of oligo(alanine) and oligo(alanine–glycine) β-sheets.^243^ Phonons were found to be reflected at the interface between the two types of residue, suggesting that heterogeneity in silkworm silk’s amino acid sequence might be the reason for its lower thermal conductivity.

#### 3.4.3 Spider Webs

At the fiber scale, finite element and coarse-grained simulations have shed light on the mechanical properties of spider webs.^42,58,231,244−255^ Computer modeling allows the study of these intricate structures without web damage or disruption of a spider’s natural behavior. For instance, imaging and parallel modeling of a tangle web enabled study of the evolution of its strength and toughness over time, showing that most of the web’s mechanical performance developed between the first and second day of construction.^246^

An early finite element study showed strand breaking in an orb web has a local effect — prestressed sacrificial silk fibers limit damage propagation, making it easier for a spider to repair a damaged web.^247^ This was later shown to be enhanced by silk’s nonlinear mechanics,^248^ as atomistically parametrized finite element simulations showed that silk’s strain-stiffening reduced the spread of damage compared to perfectly elastic and elastic–perfectly plastic materials. Spider silk’s imperfect elasticity is also crucial to prevent “catapulting” a caught insect away; the tough fibers help absorb the kinetic energy instead. High projectile velocity has been shown to favor energy dissipation through radial strands, while low velocities are dissipated mostly through aerodynamic drag on the web and prey.^249−251^

Silk’s high extensibility also plays an important role in webs. Attachment discs that anchor the web to its environment, where fibers are splayed into several contacts, were investigated in a theoretical study, and an equation for adhesive force as a function of contact angle to a surface was derived.^252^ Web anchors are made from piriform silk, which lacks rigid oligo(alanine) motifs, giving the silk extreme extensibility that lets the fibers adopt the angle that optimizes this adhesive force regardless of the initial placement of the anchor by the spider. Silk’s extensibility was also shown to be important in coarse-grained simulations on silk-on-silk junctions in webs, which showed that silk’s high extensibility makes these crossover points stronger and tougher by increasing the effective strength of adhesion.^256^

Spiders’ orb webs are well-adapted for fast winds. In windy conditions, the high modulus of silk fibers is essential for maintaining web integrity, preventing permanent deformation.^248^ Coarse-grained numerical models investigated web design under different loading conditions, and were verified by experiments on 3D-printed poly(dimethylsiloxane) orb webs.^253^ A homogeneous distribution improved localized loading, but stronger radial threads compared to capture spirals — as found in natural orb webs — improved resilience under distributed loading, such as high wind speeds. *Nephila* spiders initially weave a nonsticky spiral when constructing their webs, reducing the effective capture area. Finite element analysis has shown that fibers in these spirals are highly prestressed, making these large webs resilient to wind loading and less likely to collapse and stick onto themselves.^42^ Therefore, a trade-off between prey capture and mitigating wind damage may have evolved, a theory supported by observations that spiders reduce the densities of their webs in high winds.^250^

In addition to two-dimensional orb webs, three-dimensional tent webs from *Cyrtophora citricola* spiders have been studied; coarse-grained bead–spring simulations showed the strength and toughness of these webs increases with density, and that web resilience results from redundancy and silk’s nonlinear mechanics.^254^ The web’s outskirts were suggested to decelerate projectiles and deter predators, while funnelling prey into the dense region where the spider dwells. A neural network, “WebNet”, has recently been developed to predict the mechanical properties of these tent-like webs from variables including average fiber length, orientation, and web density.^255^ This model could be developed to solve the inverse problem — predicting the microscale factors that would yield desired mechanical properties — for use in material design.

**Figure 11:**
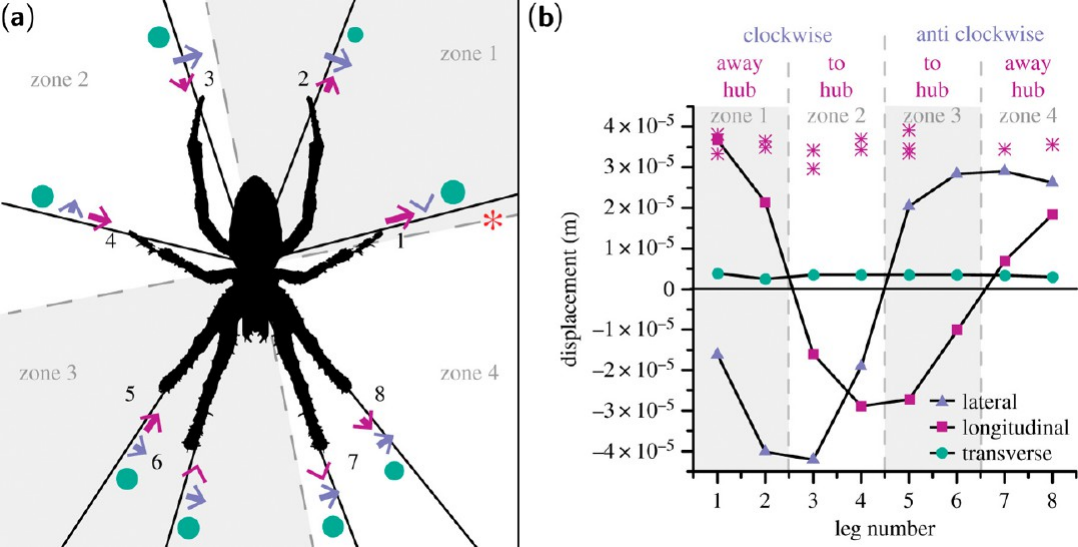
Orientation information for a hub-dwelling spider, such as *A. diadematus*. (a) Single input location (red asterisk gives radial thread, input is 83.6 mm from hub) and eight output locations at each leg based on the characteristic stance of *Araneus* at the hub for longitudinal (dark magenta; within radial axis), lateral (lilac; within plane axis, not within radial axis) and transverse (dark teal; circle) waves. Direction and relative amplitude of first peak are given by arrow direction, line length and circle diameter. The directions of the three types of waves break the web up into four zones, going anticlockwise from vibration source, indicating a rotational mesh mechanism. These zones are correct for an input polar angle of 30° with respect to the *Z*-axis — i.e. 30° bias away from hub measured from the *Z*-axis (perpendicular to web plane). (b) Longitudinal (dark magenta squares), lateral (lilac triangles) and transverse (dark teal circles) first peak amplitude varies over the eight legs at the hub for the applied input position and angle, and longitudinal first peak amplitude remains an indicator of vibration source direction as input location moves around the web (dark magenta stars), where distance to the hub is kept constant. Reproduced with permission from ref (58). Copyright 2019 the Royal Society.

Another area of study is web vibrations, which help spiders detect predators and prey. A finite element study modeled longitudinal waves, which oscillate parallel to the fiber axis, and transverse waves, which oscillate out of the orb web plane.^231^ Higher fiber stiffness increased the speed of longitudinal waves, while higher prestress accelerated transverse waves. A subsequent study investigated how vibrational information can be used by a spider to detect the site of impact on a web.^58^ A single sensor at the web’s center could not locate the source of a vibration, but a hub-dwelling spider could, by comparing differences in wave amplitudes between its eight legs. Differences in longitudinal amplitudes provided enough information to orient a spider in the direction of the source, as shown by Figure 11, and differences in transverse wave amplitudes correlated with the distance to the site of impact. A more recent study has found that prey localization is further optimized by silk fibers’ nonlinear strain-stiffening.^257^ This deeper understanding of the web’s acoustic properties has led to the proposal to manufacture silk-based eardrum grafts with a web-like foundation,^258^ and even the development of a web-based instrument.^259^

## 4 Silk-Based Materials

Over the past few years, a deeper understanding of the hierarchical structure and biological processing that give natural silk fibers their properties has enabled the design and development of innovative functional materials. In this section, we highlight some recently reported silk-based and silk-inspired materials where modeling was key in the design process or in understanding the material’s properties.

Silk from *B. mori* has been applied broadly,^260^ from supporting palladium catalysts, to use in water filtration systems and semiconductor nanopatterning.^261,262^ However, most applications of silk fibroin have been biomedical, where its biocompatibility and biodegradability are key. These properties have been studied with atomistic MD, where strong hydration of backbone amides and hydroxyl groups was suggested to weaken the foreign body response,^263^ and high exposure of randomly coiled peptides to water was proposed to speed up fibroin degradation.^47^

### 4.1 Silk Processing

To fabricate *B. mori* silk-based materials, cocoon fibers must first be cleaned and broken down, either into solutions of nanofibrils or randomly coiled protein molecules. Regenerated silkworm silk is usually obtained by first degumming cocoons to remove sericin, followed by fiber dissolution and solution purification by centrifugation and dialysis.^25^

The first step in silk processing, degumming, is often achieved by heating fibers in Na_2_CO_3_ solution.^4^ However, this can damage and reduce the molecular weight of fibroins, prompting interest into enzymatic degumming strategies.^264^ Bioinformatics analysis has recently shown that the trypsin protease is closely related to the cocoonase enzyme produced by silkworms in their final stages of metamorphosis, which breaks down cocoons. Trypsin cleaves peptide bonds adjacent to the basic amino acids arginine and lysine, common in sericin. The performance of trypsin and other proteases has since been confirmed in an experimental study, which showed the enzymes damaged fibers less than Na_2_CO_3_ treatment.^265^

After degumming, the dissolution of silk fibers is usually achieved with potent solvents such as formic acid, hexafluoroisopropanol (HFIP) and trifluoroacetic acid (TFA), with or without the application of ultrasonication.^266−268^ The mechanism of ultrasonication was studied with a coarse-grained dissipative particle dynamics approach, modeling ultrasound as a sinusoidal pressure perturbation.^269^ Ultrasound was shown to pump water into the hydrophilic regions, but not the hydrophobic regions, of the fibrillar bundle, leading to exfoliation along the fiber axis and the release of nanofibrils. An aqueous processing technique to break silk down beyond nanofibrils has been reported, where treating silk in urea and NaOH solution was shown to produce flat silk “nanoribbons” with average thicknesses of 0.4 nm.^223^ MD simulations were used to support the proposed two-dimensional structure of the nanoribbons, as the mean force required to break hydrogen bonds between fibroin chains was 40% higher than the dispersion interactions holding β-sheets together in a nanocrystal. This implies that a silk nanofibril can be thought of as a stack of nanoribbon building blocks, where the β-sheets associate to form the nanofishnet structure of β-crystallites linked by flexible regions.

Alcohols such as ethanol and methanol are regularly used in silk-based materials to promote β-sheet formation.^4,270^ Atomistic simulations have clarified the effect of these solvents on silk, showing that they promote formation of an ordered structure by interacting with polar amino acids, weakening protein–solvent hydrogen bonds so more protein–protein hydrogen bonds form.^271−273^ The reduction in solvent–protein hydrogen bonding due to alcohols’ lower dielectric constants also increases the ease of protein chain movement, leading to faster β-sheet formation than in water.^271,272^ Ethanol’s rapid induction of β-sheet formation is not always favorable in the production of silk-based materials. In an atomistic study of the self-assembly of a fibroin hydrogel, the ethanol solvent induced faster β-sheet formation than poly(ethylene glycol), making it difficult to form a low density cross-linking network.^274^

Nucleation of β-sheet formation is not a unique property of alcohols, but a general phenomenon in self-assembly. The liquid crystal and micellar models explain how solid silk fibers arise from the fibroin dope in silk-spinning animals but cannot adequately explain the self-assembly of nanofibrils in the absence of the biological spinning apparatus. The nucleation–elongation model provides a framework to understand this.^275^ Silk fibroin molecules move together randomly by thermal fluctuations and may overcome the free energy barrier to form a small β-sheet. This structure then acts as a nucleus for further, rapid β-sheet elongation, before this nucleation–elongation process repeats between β-sheets to form β-crystallites.^179^ Driven by nucleation, the self-assembly of silk proteins is therefore highly dependent on the presence of heterogeneity in solution, which lowers the free energy barrier to hierarchical structure formation. This has been observed experimentally, as the presence of graphene nanosheets greatly increases the spontaneous formation of β-sheets and nanofibrils.^276,277^

### 4.2 Chemical Modification

Chemical modifications to the surfaces of silks have been used to modulate their properties.^278^ For instance, treating *B. mori* cocoon fibers with SF_6_ plasma improves their hydrophobicity.^279^ The mechanism behind this surface treatment was investigated with DFT on a simple alanine–glycine dipeptide model of silk.^280^ The most favorable reaction pathway was the replacement of one of alanine’s methyl hydrogen atoms with fluorine via a radical abstraction mechanism. This produced C–F bonds on a Teflon-like surface, which MD simulations confirmed was more hydrophobic than natural silk.^281^ Besides SF_6_ plasma treatments, covalent chemical modifications to silks have remained largely unexplored by simulation, but some studies have investigated the noncovalent adsorption of chemicals to silks.

The most widespread use of *B. mori* silk is in textiles, where its surface is commonly treated with dyes. Due to the growing demand for “eco-friendly” dyes, the interactions between natural pigments and silk have become a subject of investigation for computational studies.^282−284^ For instance, the binding of vibrant red pigments from *Rubia tinctorum* roots to silk was studied with MD, showing that these dyes were bound by glutamate residues and that Al^3+^ ions increased the binding stability.^283^ This improvement in binding due to the presence of cations was in agreement with a previous theoretical study, where modeling the diffusion of dyes from methanol-treated silk films showed faster diffusion by more negatively charged dyes.^270^ Another natural red dye is laccaic acid, produced by *Kerria lacca* insects. The binding of these dyes to silk can be strengthened using chitosan, a carbohydrate that also stabilizes silk nanoparticles.^285^ The mechanism for this improved dye binding has recently been investigated with MD,^284^ which found chitosan’s acetylglucosamine moieties interacted strongly with silk fibroin and increased the free energy benefit from binding laccaic acid dyes.

Silk fibroin nanoparticles have emerged as a promising platform for drug delivery due to their biocompatibility and capacity for functionalization.^286^ Molecular docking studies on silk nanoparticles have shown strong binding of molecules such as curcumin, held in place by strong π–π interactions,^287^ and the chemotherapeutic drug doxorubicin.^288^ The binding of the latter drug to *B. mori* silk fibroin had previously been studied with well-tempered metadynamics, where the N-terminal domain was found to act as a pH-dependent switch for drug binding.^289^ Binding was strongest at pH 7.4, where glutamate residues were negatively charged, suggesting that modulation of the number of ionizable groups in silk could be a route to improved silk-based drug delivery systems. Glutamate residues were also shown to be important in a recent study on inhalable silk nanoparticles.^290^ Modeling showed that protonation of glutamate around pH 4 promoted favorable interactions with a bound antibiotic, increasing its antibacterial activity compared to that of the free drug.

Liposomes are also important drug delivery systems due to their excellent biocompatibility and capacity to fuse into cell membranes.^286^ Liposomes have recently been used to covalently modify azide-functionalized silk surfaces by “click” chemistry, which improved the stability of the liposomes and mitigated the foreign body response.^291^ Data from MD simulations suggested that this improvement to biocompatibility was due to the covalent click treatment improving the strength of liposome binding to silk, thereby accelerating the fusion of liposomes into lipid bilayers which resemble natural cell membranes.

### 4.3 Material Morphologies

Silk-based materials have been fabricated in diverse morphologies, such as gels, films and fibers. Modeling has facilitated the design of these materials and provided theoretical explanations of their functionalities.

#### 4.3.1 Gels and Films

Hydrogels are polymer networks that can absorb and retain large quantities of water relative to their mass, with flexibility and biocompatibility that motivate biomedical applications. Hydrogel scaffolds made from silk fibroin show promise as treatment strategies in regenerative medicine due to their mechanical performance, biocompatibility and versatility of functionalization.^292^ Modeling across length scales has provided insight into silk-based gels and films, such as explaining stability differences between gels formed from various ionic liquids,^293^ predicting the kinetics of drug release,^294^ and providing evidence that scaffolds accelerate wound healing.^295^

Pristine silk fibroin hydrogels with very high elastic moduli have been synthesized in a “binary solvent-induced conformational transition” procedure.^296^ Silk fibroin was first dissolved in HFIP before water was added, triggering the gelation mechanism and forming a structure cross-linked by β-sheets. Simulations with REMD were conducted to understand this mechanism. The low dielectric environment of HFIP solution aided formation β-sheet formation, which was then enhanced by addition of water, leading to a 40% increase in protein–protein hydrogen bonds. The proportion of β-sheets formed via the binary solvent-induced transition — dissolving first in HFIP, then adding water — was over twice that of the native protein in aqueous solution.

Structure–property relationships in *N. clavipes* silk films have been investigated in atomistic studies, where higher β-sheet content was linked to increased crystallinity and strength.^45,50^ Structure–property relationships that have been understood through modeling natural silk have also served as inspiration for the synthesis of silk-based films. These films exploit silk’s geometric confinement of its crystalline regions to impart simultaneously high strength and toughness.^203^ Graphene oxide quantum dots, representing β-crystallites, held together poly(vinyl alcohol) flexible linkers by hydrogen bonds, giving the thermally stable films high strength of over 150 MPa.

Four-dimensional (4D) printing is the use of 3D printing technology to generate responsive materials and has recently been used to make a biocompatible silk fibroin hydrogel.^8^ Finite element analysis modeled the effect of structural changes on silk fibroin hydrogels upon swelling with a cell culture solution, and a flat hydrogel that would curl up as it swelled was designed. As the modeling predicted, the 3D-printed hydrogel transformed into a curved shape as it swelled, ready for implantation into a rabbit’s damaged trachea where it integrated naturally with a low immune response. This exemplifies a general workflow for the development of biocompatible materials, where modeling is used to design 3D-printable gels that change, upon a stimulus, into a structure ready for implantation *in vivo*.

Unlike most proteins, which denature at high temperatures, elastin folds into a contracted form in a transformation known as the inverse temperature transition.^297^ This transition results from elastin’s GXGVP motif, where X is any amino acid other than proline, and has been observed in similar motifs found in spider silk.^298^ The fundamental cause of the transition is similar to that behind supercontraction: a stimulus allows the peptide to recoil into a more entropically favorable configuration. The inverse temperature transition is of interest for developing stimuli-responsive materials made from silk-elastin-like peptides (SELPs) which incorporate silk’s mechanical properties in GAGAGS blocks with elastin’s GXGVP blocks. REMD has been used to develop computational models of SELPs, and has shown an increase in protein–protein hydrogen bonding after the inverse temperature transition.^297^ Modeling has also guided the design of stimuli-responsive SELP hydrogels, showing that different response could be achieved by changing the identity of the “X” residue in the elastin block.^299^ For example, using glutamate conferred a temperature response at high pH which could be disabled in water.

The silk blocks in SELP hydrogels form cross-links, thereby speeding up gelation.^300^ Coarse-grained modeling has revealed that this cross-linking can limit the extent of the contraction at high temperatures, as intermolecular constraints trap the molecules in higher energy states.^301^ A recent study, combining atomistic and coarse-grained MD with experiment, investigated structural changes in response to ion concentration and temperature in a SELP molecule and a SELP where tyrosine residues were coupled to a diazonium salt.^302^ This diazonium coupling reduced the number of exposed dityrosine cross-link sites, slowing down gelation and attenuating the response of the SELPs to heat and ions.

#### 4.3.2 Fibers

The production of fibers that can match or exceed the mechanical performance of natural silk is a major area of research.^9^ Several bioinspired silk spinning methods to transform an isotropic silk fibroin solution into strong and tough fibers have been developed, often guided by fluid dynamics simulations.^134,303,304^

Electrospinning is one such technique, which generates ultrafine fibers by applying a high voltage to draw charged threads from solution. However, the mechanical properties of electrospun fibers often fail to meet the expected standards.^25,305^ The reason for the fibers’ reduction in strength was recently investigated in an atomistic MD study.^306^ Application of an electric field in the antiparallel direction to the β-sheet-forming region decreased silk fibroin’s elastic modulus and tensile strength, due to disruption of hydrogen bonding between β-sheets. This discovery might lead to optimization of the electrospinning process, as lower power machines that produce electric fields below 0.1 V nm^–1^ are not predicted to suffer the same problems.

Atomistic modeling has also provided guidance about general principles for fiber design. The presence of more hydrophobic domains was found to significantly increase material strength due to increased stacking of β-crystallites but lead to failure at lower strains.^307^ Conversely, fibers with many glycine-rich domains were highly stretchable but weaker. Coarse-grained simulations have studied the effect of the terminal group on a fiber-forming core domain in silk-mimetic block copolymers, finding that increasing the molecular weight and hydrophilicity of the termini improved peptide chain alignment under shear flow and promoted the formation of semicrystalline domains, leading to improvements in strength and elasticity but reductions in toughness and extensibility.^308^

**Figure 12:**
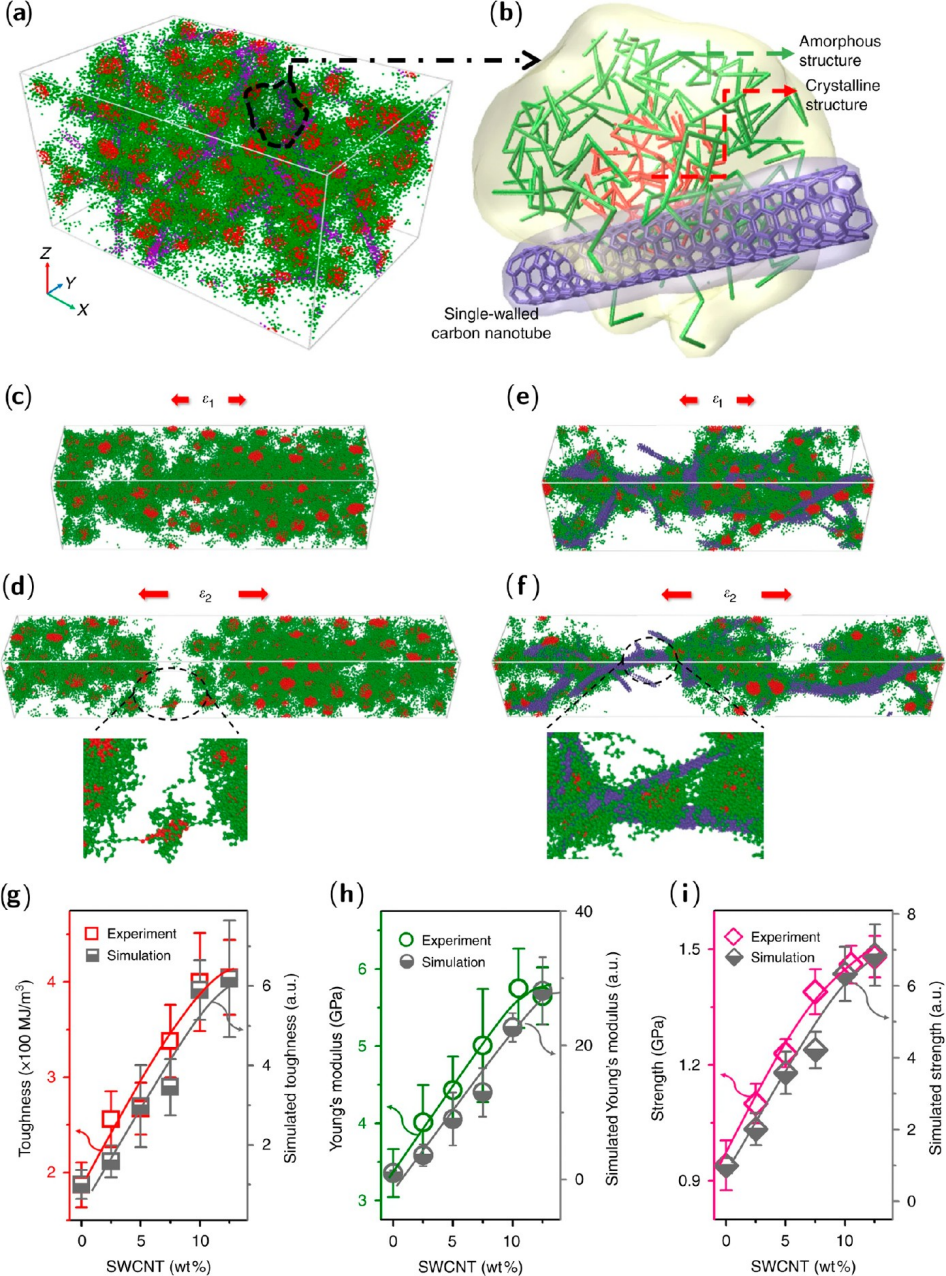
A typical mechanical test of the silk-SWCNT nanocomposite in DPD simulation. (a, b) Snapshot of the coarse-grained S-silk composite. Red: crystalline structure, β-sheet structure; green: amorphous structure (3_1_-helices and β-turns); purple: SWCNT. (c–f) DPD-simulated images showing the structural evolution of natural S-silk (c, d). S-silk composite@10% SWCNT (e, f). Along the *x*-axis with increasing strain, ε_1_ < ε_2_. ε_2_ is the critical strain at which spider silk is broken. Because of the hydrophobic interactions between SWCNT and spider silk, the S-silk composite has better mechanical properties than natural S-silk. (g–i) Graphs showing toughness, Young’s modulus and strength increased with increasing wt % of SWCNT, indicating that SWCNT is critical for improving the mechanical properties of the S-silk composite. DPD simulation and experiments agree well. The error bars of g–i for experiments show standard deviations based on 50 independent samples. The error bars of g–i for simulations show standard deviations based on 5 independent simulations. Reproduced with permission from ref (309). Copyright 2020 Springer Nature.

Recently, an electrically conductive tendon based on spider silk has been created for the development of tendon-driven robotic hands, with extremely high toughness of 420 MJ m^–3^.^309^ Single-walled carbon nanotubes — which had previously been fed to silkworms to produce tougher biologically spun fibers^310^ — were used to toughen the silk, creating fibers that could simultaneously transmit force and electrical signals between sensors and actuators. To understand how these single-walled carbon nanotubes improved silk fibers’ mechanics, dissipative particle dynamics simulations were performed on the model shown in Figure 12. The effect of nanotubes on mechanical properties was predicted in good agreement with experiment and shown to result from strong hydrophobic interactions. Such interactions were also shown to improve mechanical properties in a study of carbon nanotube fibers infiltrated with silk fibroin, where addition of the silk improved strength and toughness by 250% and 130% respectively.^311^

### 4.4 Composites

Combining silks with other materials can yield composites that integrate the desirable properties of each constituent.^312,313^

Computational studies have been instrumental in understanding the interactions between materials, such as in composites of silk with epoxy and collagen, where modeling shed light on the materials’ binding and degradation.^314−318^ Atomistic studies have also provided guidance on the creation of composites. For instance, an MD investigation found that alanine and glycine in isolation can “glue” cellulose strands together as well as flagelliform silk can, and that alanine acts as a better bioglue when dry while glycine performs better when wet.^319^ Modeling has also been used to study the self-assembly of composites — a coarse-grained study on collagen–silk–collagen polypeptides showed that self-assembly into a “β-roll” structure is a nucleated process, requiring the formation of trimeric or tetrameric structures.^79,320^

#### 4.4.1 Biomineralization

The process by which organisms produce mineralized tissues such as bones, teeth and shells from biomacromolecular templates is known as biomineralization.^321^ Silks can be used as such templates, and their biomineralizing capabilities have been the subject of several computational studies. For instance, modeling predicted the “honeycomb” structure of an osteoinductive silk–TiO_2_ composite,^322^ and showed that the crystallization of an unstable polymorph of CaCO_3_ on *B. mori* silk resulted from geometric complementarity between the mineral and silk fibroin’s amide backbone.^323^ However, most studies have focused on biomineralization of crystals such as hydroxyapatite, which makes up bones and teeth, and silica, which constitutes the shell-like cell walls of diatoms.

The potential for synergy between simulation and experiment was clearly demonstrated in studies of a silk-based protein that induces silica nanoparticle formation. This protein was designed by combining *N. clavipes* dragline silk’s repetitive region with a silica-promoting peptide, and recombinantly expressed to generate films that promote the growth of silica nanoparticles.^324^ REMD was used to predict the folding of this chimeric protein, and its silica-promoting ability was suggested to result from the exposure of positively charged amino acids on the protein’s surface. The silica nanoparticles generated by the films were shown by MD to activate a particular integrin which mediates the differentiation of human mesenchymal stem cells into bone-synthesizing osteoblasts, and this differentiation was confirmed experimentally.^104,325^ Recently, osteoinduction was shown to be optimal for silica nanoparticles around 200 nm in size, which MD data suggest results from favorable electrostatic interactions between integrins and nanoparticles at this length scale.^49^

Atomistic MD has also been used to study the interactions between *B. mori* silk domains and a hydroxyapatite surface.^326^ While the structures of the amorphous and N-terminal domains were largely unaffected by binding, silk fibroin’s crystalline domains experienced a substantial reduction in β-sheet content, suggesting that formation of silk–hydroxyapatite composites might require sacrifice of some of silk’s mechanical properties. The binding was later investigated by a larger model of linked crystalline and flexible regions, and shown to be driven by the interactions between silk’s negatively charged amino acids with calcium ions,^327^ becoming stronger as hydroxyapatite content increased.^328^

Silk’s propensity for biomineralization has been exploited in the design of a multilayered nanoporous membrane for water filtration.^261^ Coarse-grained simulations of protein nanofibrils and mineral plates revealed that formation of a segregated, multilayer structure required a weak interaction between the protein and mineral constituents. This insight motivated the choice of silk nanofibrils and hydroxyapatite mineral plates as the building blocks, which were combined to synthesize a highly purifying porous membrane. The membrane’s soft silk layer had small pores that filtered out contaminants effectively, while the hard mineral layer enabled fast water penetration and provided structural support. The biomineralization of silk with hydroxyapatite has also been exploited by mixing the mineralized solution with chitin nanofibrils.^329^ Coarse-grained bead–spring modeling of these three materials showed that chitin nanofibrils improved the composite’s strength and toughness, which was later confirmed by experiment. These examples highlight the potential for the computational rational design of biomaterials before experimental synthesis and validation of mechanical properties.

#### 4.4.2 Bioelectronics

There is great interest in developing conductive and flexible biomaterials for healthcare.^330−334^ Atomistic simulations have shown that silk fibroin can be combined with conductive materials such as biobased carbon without loss of strength, to create highly stretchable and biocompatible materials made from renewable sources.^333^

Atomistic MD has elucidated mechanisms by which silk’s plasticity can be increased to make stretchable hydrogels. Studies have shown that the addition of CaCl_2_,^330^ and tannic acid,^331^ to *B. mori* silk increases its extensibility by preventing the formation of hydrogen bonds between silk fibroin chains, thereby decreasing the proportion of β-sheets and increasing random coil formation. A similar effect was observed in MD simulations by the addition of glycerol, which was used to tune the properties of silk fiber mats, improving water permeability to make sweat-tolerant electrodes to interface sensors and the skin with high signal fidelity.^332^

Recently, small molecules that could functionalize silk scaffolds to bind integrins on the neuronal cell surface were predicted using machine learning, suggesting possible designs for functional silk scaffolds.^67^ Transparent silk fibroin hydrogels may also be useful to study the brain, enabling simultaneous electrical and optical analytical techniques to be used. A transparent electrode based on silk fibroins cross-linked by poly(ethylene glycol) has recently been developed.^334^ This cross-linking increased the elastic modulus from 1.5 to 10.7 MPa and the extensibility by 400% compared to pristine silk hydrogels, which atomistic simulations suggested resulted from a decrease in β-sheet content. The cross-linking also increased the solvent-accessible surface area, which might improve the gels’ biocompatibility as the hydration layer could prevent the binding of antibodies.

**Figure 13:**
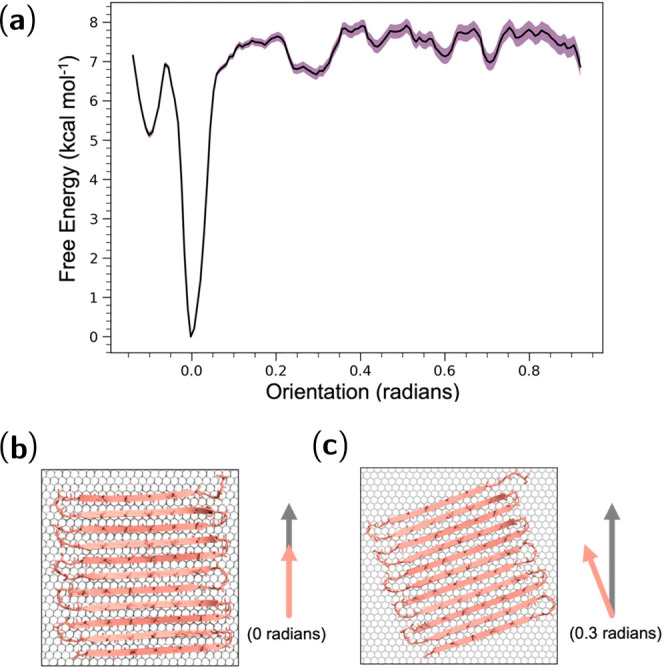
(a) Free energy of a single silk fibroin protein as a function of orientation to the armchair edge of graphite with error bars drawn. (b) Silk fibroin orientation at 0 radians. This is the most stable configuration and corresponds to protein alignment along the armchair edge of graphite. (c) Silk fibroin at 0.3 rad, a metastable state in which the protein is more closely aligned to the zigzag edge of graphite. Reproduced with permission from ref (336). Copyright 2024 American Chemical Society.

The hydrophobic surfaces of graphite and graphene are electrically conductive and readily functionalizable, so of great interest for materials design and incorporation into silk-based composites.^335−337^ The mechanism of silk nanofibril assembly on graphene has recently been studied with plasmonic infrared spectroscopy and MD simulation.^335^ Graphene was shown to nucleate β-sheet formation, before these secondary structures connected with nearby molecules and grew into rod-like nanofibrils, which aligned and elongated. In addition to nanofibrils, a highly ordered two-dimensional layer of silk has recently been reported to form on a graphite surface, growing layer by layer upon increasing fibroin concentration.^338^ The thermodynamics of this lamellar structure were studied by MD simulations using umbrella sampling.^336^ To form the two-dimensional layer, the alanine residues of *B. mori* silk must face away from the hydrophobic surface to enable the small glycine residues to pack closely and expel water at the interface between materials. Figure 13 shows that there was a clear free energy benefit for silk fibroin β-sheets to align directly along the “armchair” axis of graphite, in a highly ordered lamellar structure.

The interactions of graphene with preassembled silk fibroins have also been studied by MD.^337,339^ In silk fibroin, binding to graphene was shown to weaken protein–protein interactions and reduce β-sheet content in the crystalline fractions, while increasing the stability and helical content of the flexible regions.^337^ Graphene oxide has been shown to cause less disruption than pristine graphene to silk fibroin’s structure due to diminished hydrophobic interactions.^340^ Another study showed that the oxygen atoms in graphene oxide act as important hydrogen bond acceptors and investigated the effect of water content on silk–graphene oxide composites.^341^ At low relative humidity, water was shown to help the formation of hydrogen bonds between graphene oxide and silk, but when hydration became too high, water lubricated the surfaces and reduced the composite’s strength.

## 5 Future Perspectives

Drawing inspiration from nature’s engineers has enabled the development of innovative functional materials. Advancements in computational power, algorithm efficiency and software usability have made modeling an indispensable tool for materials science to predict properties and understand structure–function relationships.

Remarkably, in just two decades, the state of the art in simulations on silks has progressed from calculating trajectories of simple model peptides to fully atomistic stress–strain analyses of nanofibrils. The early focus of modeling involved refining silk structures and understanding the basic engineering principles behind fibers’ mechanical properties. Since then, atomistic and coarse-grained studies have elucidated silk fibers’ self-assembly and evolutionary refinement, and finite element studies have explained how spider silk’s nonlinear mechanics produce remarkable emergent properties in webs.^248^ These studies have provided understanding into natural silks, enabling the development of strong, tough and functional biomaterials such as water-activated artificial muscles,^10^ 4D-printed hydrogels,^8^ and electrically conductive tendons.^309^

Despite these advancements in modeling, simulations are inherently limited. Discrepancies between secondary structures predicted by MD simulations and experimental spectroscopic data may suggest force field limitations. Quantum mechanical methods have thus far been intractable for use on silk proteins due to their steep scaling with respect to system size, but due to the development of linear scaling methods,^342−344^ DFT optimizations of silks’ secondary structures to validate force field methods might become viable in the near future.

On a larger scale, the recent development of an optimized coarse-grained force field for silk^97^ should enable accurate simulations over long scales of length and time. These simulations might reveal more about the self-assembly processes of antiparallel β-sheets in β-crystallites, and explore the influence of changes in solution composition throughout the spinning duct. Recent discoveries suggest that low molecular weight components like the spider silk constituting element (SpiCE) protein, which simulations show improve fibers’ elastic modulus through salt bridge formation and hydrogen bonding, are partially responsible for spider silks’ mechanical properties.^345,346^ So far, the focus of MD simulations on spider silk has been on MaSp1 and MaSp2, but future coarse-grained studies might investigate the interactions between these proteins with other proteins present in some spider silks, including MaSp3, SpiCE and cysteine-rich proteins.^345,347^ Spider silks other than those from the major ampullate glands may also see greater focus, such as aciniform silk which spiders use to wrap prey, for which an α-helix-rich structural model has recently been refined using AlphaFold.^348^

Over the last five years, the most significant development in modeling silks may have been the emergence of machine learning models. A recently developed model, “ForceGen”, can generate protein structures to fulfill mechanical property design objectives, allowing exploration of mechanobiological space beyond the constraints of biological synthesis.^349^ Models such as ForceGen not only allow the prediction of structures, but can also improve our understanding of structure–function relationships which are transferable between biological materials. Presently, there is a limited set of data on silk proteins’ mechanical properties, but since data can be generated by SMD simulations, increasing computational power will allow the curation of larger training sets. Machine learning models are therefore set to become more powerful tools for materials design. Multiscale modeling of natural silks and silk-based biomaterials is an ever-growing field that faces challenges and exciting opportunities. Effective use of modeling from the molecular to fiber scale promises to further improve our understanding of natural silk fibers, and assist in the design and optimization of robust, biocompatible and functional biomaterials.

## Abbreviations

DFT: density-functional theory
MaSp: major ampullate spidroin
MD: molecular dynamics
NMR: nuclear magnetic resonance
REMD: replica-exchange molecular dynamics
SELP: silk-elastin-like peptide
SMD: steered molecular dynamics
SWCNT: single-walled carbon nanotube
